# Schistosome sex matters: a deep view into gonad-specific and pairing-dependent transcriptomes reveals a complex gender interplay

**DOI:** 10.1038/srep31150

**Published:** 2016-08-08

**Authors:** Zhigang Lu, Florian Sessler, Nancy Holroyd, Steffen Hahnel, Thomas Quack, Matthew Berriman, Christoph G. Grevelding

**Affiliations:** 1 BFS, Institute of Parasitology, Justus-Liebig-University, Giessen, Germany; 2 Wellcome Trust Sanger Institute, Wellcome Genome Campus, Hinxton, United Kingdom

## Abstract

As a key event for maintaining life cycles, reproduction is a central part of platyhelminth biology. In case of parasitic platyhelminths, reproductive processes can also contribute to pathology. One representative example is the trematode *Schistosoma*, which causes schistosomiasis, an infectious disease, whose pathology is associated with egg production. Among the outstanding features of schistosomes is their dioecious lifestyle and the pairing-dependent differentiation of the female gonads which finally leads to egg synthesis. To analyze the reproductive biology of *Schistosoma mansoni* in-depth we isolated complete ovaries and testes from paired and unpaired schistosomes for comparative RNA-seq analyses. Of >7,000 transcripts found in the gonads, 243 (testes) and 3,600 (ovaries) occurred pairing-dependently. Besides the detection of genes transcribed preferentially or specifically in the gonads of both genders, we uncovered pairing-induced processes within the gonads including stem cell-associated and neural functions. Comparisons to work on neuropeptidergic signaling in planarian showed interesting parallels but also remarkable differences and highlights the importance of the nervous system for flatworm gonad differentiation. Finally, we postulated first functional hints for 235 hypothetical genes. Together, these results elucidate key aspects of flatworm reproductive biology and will be relevant for basic as well as applied, exploitable research aspects.

Schistosomiasis is a water-borne infectious disease caused by platyhelminths of the genus *Schistosoma*. More than 780 million people live in endemic areas of Africa, Asia, and South America and an estimated 230 million people are infected with one of the six *Schistosoma* species relevant for humans. *Schistosoma mansoni, S. japonicum, S. mekongi, S. guineensis* and *S. intercalatum* cause intestinal schistosomiasis, whereas *S. haematobium* initiates urogenital schistosomiasis^1^. Morbidity and mortality are high for schistosomiasis, which is among the most severe parasitic diseases affecting human, second only to malaria. Travelers, migrants, and immigrants are also affected, which is an emerging problem in industrialized countries^2^. Concern has recently increased even in Europe because of the sudden re-emergence of urinary schistosomiasis in Corsica, France^3^. Schistosomes also infect animals including livestock and thus have an additional socio-economic impact. These animals include water buffaloes transmitting *S. japonicum*, which causes one of the most prevalent zoonoses in Asia^4^.

The schistosome life cycle includes an intermediate water-snail host, where the infectious larval stage (cercaria) develops. After shedding from the snail, cercariae swim through the water and penetrate the skin of a vertebrate host to transform into schistosomula. They migrate via the blood circulation to the portal vein to become adults with separate sexes. Dioecy is a defining characteristic of schistosomes, which belong to an otherwise hermaphroditic phylum^5^. During passage through the liver, male and female schistosomes pair, leading to the maturation of the female reproductive organs. The constantly paired couples migrate to their final destinations in the mesenteric veins of the gut (most species) or the urinary venous plexus of the bladder (*S. haematobium*)^1^. After pairing, egg production is initiated and results in the daily production of 300–3,000 eggs per female, depending on the species^6^. Approximately half of the eggs reach the gut lumen (most species) or the bladder (*S. haematobium*). The remaining eggs are swept away via the blood system mainly into liver and spleen, where they penetrate the tissues causing severe inflammation and liver cirrhosis, the main cause of mortality^1^.

There is only one drug, Praziquantel (PZQ), to treat schistosomiasis, a vaccine is not yet available, and resistance may be emerging. Therefore, new drug targets are needed^7^. Due to the importance of eggs for the life cycle and for inducing pathogenesis, past studies have focused on the identification of genes controlling reproductive development^8^,^9^. This was supported by genome projects that unravelled the parasite’s genetic repertoire^10^,^11^,^12^. Although first insights into processes contributing to female gonad differentiation have been obtained, especially with respect to the roles of kinases^8^,^13^,^14^,^15^, the reproductive biology of schistosomes is poorly understood. Nevertheless, the elucidation of processes controlling gonad development and egg production is critically important to discover novel strategies to combat schistosomiasis^8^,^16^.

To identify molecules and molecular mechanisms contributing to sexual maturation we therefore performed tissue-specific RNA-seq analyses of complete ovaries and testes of *S. mansoni* females and males obtained from single-sex or bisex (mixed sex) infections.

## Results

### Transcriptome analysis, a first overview

RNA was extracted from complete testes (T) and ovaries (O) of adult *S. mansoni* (Supplementary Fig. S1) obtained from single-sex (s; unpaired worms) or bisex (b; paired worms) infections using the recently established organ isolation method^17^. For comparisons, the transcriptomes of whole males (M) and females (F) were also determined. After quality filtering, 43–110 million reads (average 63 million) were obtained for each sample, and 84.5% mapped to version 5 of the *S. mansoni* reference genome (Supplementary Table S1). Pearson’s correlation analysis confirmed high reproducibility and consistent quality among the biological replicates (r > 0.82; Supplementary Table S2). Using RPKM >2 as threshold genes with low transcript values (1,049–1,897) were filtered out before subsequent analyses (Supplementary Table S3). Overviews of the top 100 transcribed genes, which included genes encoding p14, GAPDH, HSP70, α-tubulin and ribosomal proteins, are provided in Supplementary Fig. S2, Supplementary Table S4, and Supplementary Notes.

### Sample distance analyses revealed expected and unexpected relationships

**Figure 1:**
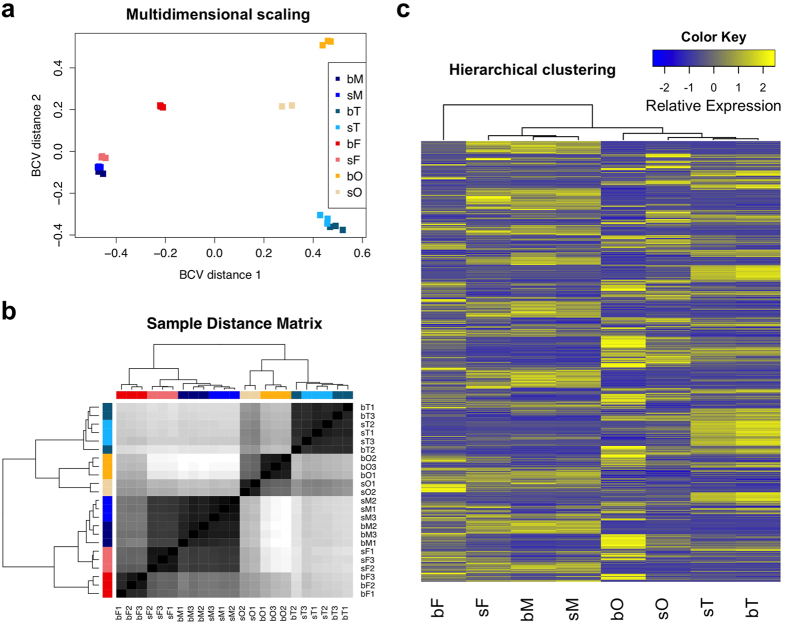
Sample relationships revealed by three different approaches. (**a**) Multidimensional scaling (replicates for each sample are indicated by the same color). (**b**) Sample distance matrix based on a black/white scale. The samples were colored the same as in (**a**). (**c**) Hierarchical clustering based on RPKM values of all genes and the scaling was done across all samples for each gene. bM, bisex (paired) males; sM, single-sex (unpaired) males; bT, testes from bisex males; sT, testes from single-sex males; bF, bisex (paired) females; sF, single-sex (unpaired) females; bO, ovaries from bisex females; sO, ovaries from single-sex females.

Multidimensional scaling and sample-distance matrix analyses showed high congruence within the replicates of the different samples (Fig. 1a,b). Furthermore, sM and bM samples occurred in one cluster, as did the sT and bT samples, indicative of highly similar transcriptomes. In contrast, sF and bF samples split in two separate clusters, as did sO and bO samples. Pairing, therefore, had a more pronounced effect on females and ovaries than on males or testes. A similar tendency was also observed in the hierarchical clustering of all genes (Fig. 1c). As expected, whole worm clusters were found to be positioned distantly from their gonad counterparts, and testis samples clustered separately from the ovary samples. However, the distance between sF and both male samples (bM and sM) was shorter than the distances between sF and bF, or bF and males.

### The effect of pairing, gender and tissue-origin on transcription

With respect to pairing effects, transcripts detected in bM, bF, bT, and bO were compared to those from sM, sF, sT, and sO. To investigate gender effects, transcripts of male worms or testis were compared to their female counterparts (bM/bF, bT/bO, sM/sF and sT/sO). To unravel tissue effects, comparisons were made between bT and bM, sT and sM, bO and bF, as well as sO and sF). (Table 1; Supplementary Fig. S3; Supplementary Table S5). For all comparisons, we used a threshold of fold change >1.5 to determine differential expression.

For genes transcribed in males and testes only 3–5% were predicted to be significantly affected by pairing even with a relatively permissive threshold (False Discovery Rate (FDR) < 0.05). In contrast, transcript levels of >43% of the genes were affected by pairing in females and ovaries (FDR < 0.005). In terms of the absolute number of differentially transcribed genes, pairing had a far greater effect on female worms and ovaries than on males and testes (Table 1). Furthermore, using multidimensional scaling, a single cluster represented the male (bM and sM) or testes (bT and sT) datasets, but the female (bF and sF) and ovaries (bO and sO) formed separate clusters (Fig. 1a). Pairing, therefore, strongly influenced transcription and the effect was particularly pronounced in the female worm and ovary.

In bisex infections 51–58% of genes were differentially transcribed between males and females (comparing whole worms or their respective gonads). However, the effect of gender on samples from single-sex infections was smaller; in particular, only 16.8% of genes were differentially transcribed between whole worms (sM and sF) (Table 1).

The effect of tissue origin, finally, seemed to be similar among the samples. In each case, more than 50% of the detected genes were significantly differentially transcribed between whole worms and gonads (Table 1).

### The effect of pairing on the gonadal transcriptomes is pronounced in females

In the testes, 243 genes were significantly influenced by pairing. Transcripts for 96 of these were more abundant in bT and 147 were more abundant in sT. However, in ovaries the number of genes affected by pairing was nearly 15-fold greater–with 3,600 genes differentially transcribed between bO and sO. Transcripts of 1,752 and 1,848 genes were more abundant in bO and sO, respectively (Table 1 and Supplementary Table S6). Based on mapping to apparent orthologs within the KEGG database, 849 of the bO genes were involved in ribosome biogenesis, RNA transport, oxidative phosphorylation, endocytosis, Akt-, MAPK-, and Ras-signaling pathways, whereas the mapped sO genes (435 orthologs) appeared to be involved in MAPK signaling, lysosome function, and focal adhesion (Supplementary Table S7).

**Figure 2:**
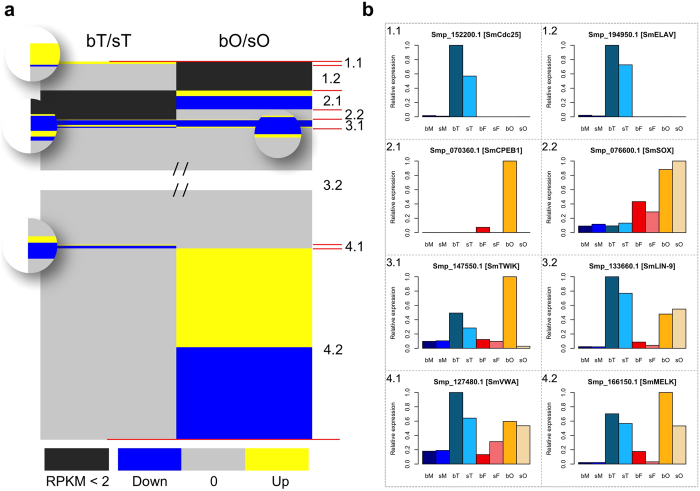
Overview of the divisions of DEGs of testes and ovaries into 8 categories (Cat) based on their tissue-preferential/specific and pairing-dependent transcript occurrence. Cat 1.1: Testis-preferential/specific and pairing-affected genes. Cat 1.2: Testis-preferential/specific and pairing-unaffected genes. Cat 2.1: Ovary-preferential/specific and pairing-affected genes. Cat 2.2: Ovary-preferential and pairing-unaffected genes. Cat 3.1: Pairing-affected genes in both gonads. Cat 3.2: Pairing-unaffected genes in both gonads. Cat 4.1: Transcripts of genes affected by pairing in testes but not in ovaries. Cat 4.2: Transcripts of genes affected by pairing in ovaries but not in testes. (**a**) Graphical illustration of the categories. The left and right parts indicate DEGs in testes (bT/sT) and ovaries (bO/sO), respectively. Grey color indicates genes that were not found to be significantly differentially transcribed. Blue and yellow areas represent genes whose transcript numbers were significantly lower or higher after pairing, respectively. (**b**) Transcript profiles of one representative gene of each category on a relative basis of transcript amounts. To provide more information about the transcript profiles of categorized genes, we added their relative expression also for the adult samples. The selected genes represent a cell-division cycle phosphatase *cdc25* (Smp_152200; Cat 1.1), *elav* (embryonic lethal, abnormal visual system) (Smp_194950; Cat 1.2), *cpeb1* (cytoplasmic polyadenylation element binding) (Smp_070360; Cat 2.1), a *sox* transcription factor (Smp_076600; Cat 2.2), a potassium channel of the *twik* (tandem of P domains in a weakly inward rectifying K^+^ channel) family (Smp_147550; Cat 3.1), *lin-9* (Smp_133660; Cat 3.2), a *von-Willebrand factor A* (vWA) domain-containing protein gene (Smp_127480; Cat 4.1), and *melk* (maternal embryonic leucine zipper kinase) (Smp_166150; Cat 4.2).

In view of gonad-specific/preferential gene expression and the effect of pairing, genes were classified into 8 categories, as follows: genes preferentially expressed in testis and either affected (1.1) or unaffected (1.2) by adult-pairing; genes preferentially expressed in ovaries and either affected (2.1) or unaffected (2.2) by pairing; genes expressed in both gonads that are affected (3.1) or unaffected (3.2) by pairing; genes affected by pairing in testes but not in ovaries (4.1); or genes affected by pairing in ovaries but not in testes (4.2) (Fig. 2 and Supplementary Fig. S4; Supplementary Table S8; Supplementary Notes).

### Compared to males the female transcriptome is influenced to a greater extent by pairing

Although no obvious morphological differences between bM and sM, including their testes, were found in the present study or elsewhere^8^,^18^, 426 genes were differentially transcribed between males that had, versus those that had not, experienced pairing (Table 1 and Supplementary Table S6). This supports our previous hypothesis on bidirectional molecular communication between the genders. Based on SuperSAGE, microarray and qPCR data, transcript profiles had been obtained demonstrating the pairing-dependent transcription of individual genes in males and testis^19^,^20^. This included SmFst (Smp_123300), a follistatin ortholog which was localized by *in situ* hybridization in the testis and found to be more abundantly transcribed in sM^19^. The latter is confirmed now by the RNA-seq data (2.72-fold difference; Supplementary Fig. S5). The higher transcript level of SmFst in sF compared to bF indicates a similar role in females. As a potential TGFβ antagonist, SmFst may be involved in controlling TGFβ-signaling, which may have an important but yet unknown role in testes and ovaries following pairing.

The comparison of female transcriptomes revealed 3,748 differentially expressed genes (DEGs). Transcript levels of 1,591 and 2,157 genes increased in paired and pairing-unexperienced females, respectively (Table 1 and Supplementary Table S6). Known schistosome egg-shell precursor genes p14 (Smp_131110) and p48 (Smp_014610) were among those most affected by pairing in females, as were the genes SmTyr1 (Smp_050270) and SmTyr2 (Smp_013540), which encode tyrosinases that are female-specifically transcribed. Their activities have been localized to the vitellarium, and they are involved in egg-shell synthesis^21^. In our analysis, only few transcripts (below threshold) of both tyrosinase genes were detected in whole males or testes. SmTyr2 was additionally transcribed in bO (above threshold; Supplementary Fig. S5).

### Pairing turns male-like virgin schistosomes into mature females

**Figure 3:**
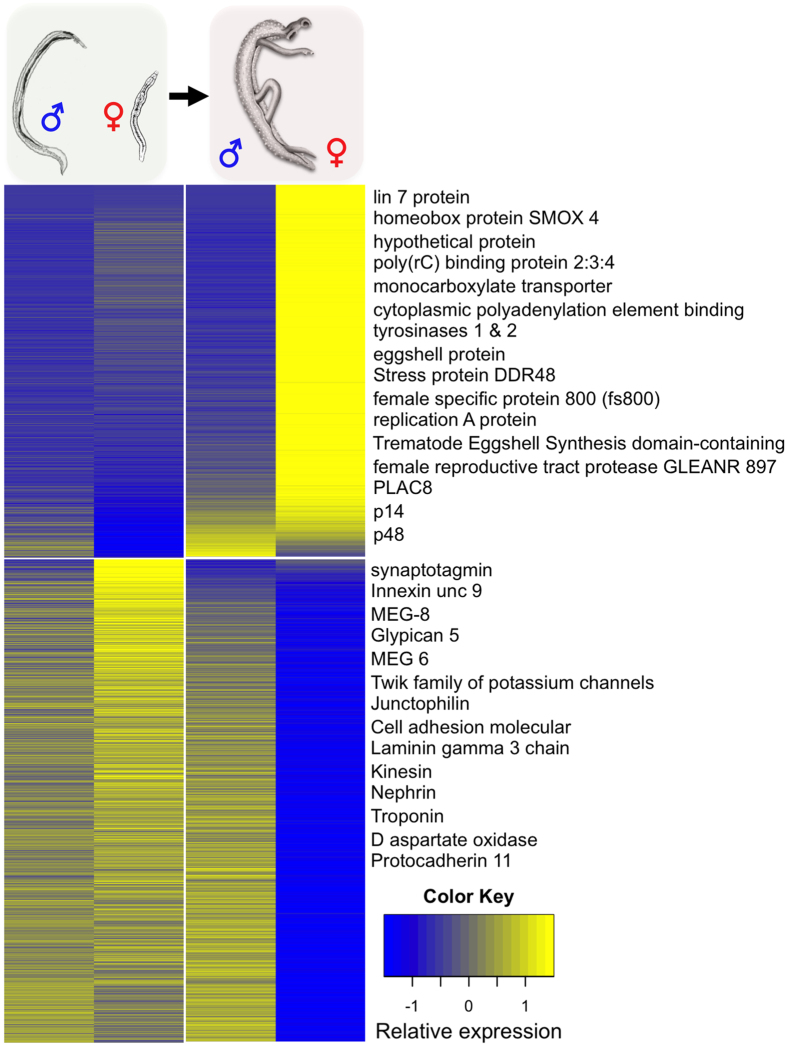
Heatmap presentation of the hierarchical clustering of 3,748 female DEGs based on their higher (upper panel, 1,591 genes) or lower (lower panel, 2,157 genes) transcript numbers. Transcript abundance across all worm samples is shown as follows: sM (1. column), sF (2. column), bM (3. column), and bF (4. column). Yellow and blue colors indicate high and low abundance, respectively. Exemplary genes (comprising largest fold-changes) are indicated by name showing that e.g. egg synthesis-associated genes such as tyrosinase, p14, and p48 are transcribed at the highest levels in bF (upper panel). In contrast, transcript levels of genes associated with motor function such as kinesin or troponin, or cell adhesion such as cell adhesion molecular or protocadherin decreased in bF (lower panel).

Compared with females from bisex infections, multidimensional scaling (Fig. 1) provided evidence that the transcriptome of single-sex females is remarkably more similar to those of males. Is there a closer biological congruence between the sF and males? To address this issue an attempt was made on the basis of female DEGs (Supplementary Table S6) to comparatively analyze their transcript profiles in sF versus males and bF versus males. A striking shift in the transcript patterns was observed in the female after pairing (Fig. 3), which indicates a functional transformation. More than 77% of female DEGs (2,912 out of 3,748) showed significant differential transcript occurrence between bF and bM. Only 21.4% (801 out of 3,748) differed between sF and sM. This indicates that before pairing the female is more similar to a male with respect to gene expression. The top 50 genes with respect to transcript abundance in bF included representatives with proven roles in egg biosynthesis, including the tyrosinases SmTyr1 and SmTyr2, as well as the eggshell precursor proteins p14, p48, and fs800, whose transcript levels increased ≥43.7-fold after pairing. Furthermore, two *cpeb* genes (Smp_070360, Smp_137460) were detected, which fulfill potential roles in oocyte maturation^22^. The occurrence of genes with reduced transcript levels in bF may suggest that specific functions are abandoned after pairing. Given that 78.7% (1,699 out of 2,157) of these genes did not show significantly differential transcription between males and sF (Supplementary Table S9), they might be representatives for common functions. Among these are motor function-associated genes, cell adhesion molecules (Fig. 3), and according to GO analysis also genes involved in signal transduction and ion transport (Supplementary Fig. S6).

### Identification of genes with high transcript abundance in both testes and ovaries compared to whole worms

By combining all genes whose transcript levels were higher (≥1.5-fold difference) in all reproductive organs compared to whole worms, a gonad-biased transcription was found for 1,012 genes across all four groups (Supplementary Table S10). GO enrichment analysis was performed (Supplementary Fig. S7) based on the categories “biological process” and “molecular function”. The main processes identified included RNA biosynthesis, cell cycle, DNA replication, DNA repair, chromosome organization, nuclear division, ribosome biogenesis and mitosis. About half of these genes (557 out of 1,012) have binding activities including nucleic acid binding and ATP binding. Furthermore, 63 gene products have ATPase activity, 49 possess helicase activity, 48 exhibit motor activity, 39 actin-binding properties, and 32 have calmodulin-binding activity. All the categories were exactly what we expected to find for gonads. Beyond that we detected 235 genes coding for “hypothetical proteins” and without conserved domains (E-value cutoff: 0.01). Due to their gonad-preferential transcription it seems likely that these genes represent novel players in reproduction-associated processes in *S. mansoni* (Supplementary Table S10).

### Stem cell- and neoblast-associated genes are transcribed in the gonads

**Figure 4:**
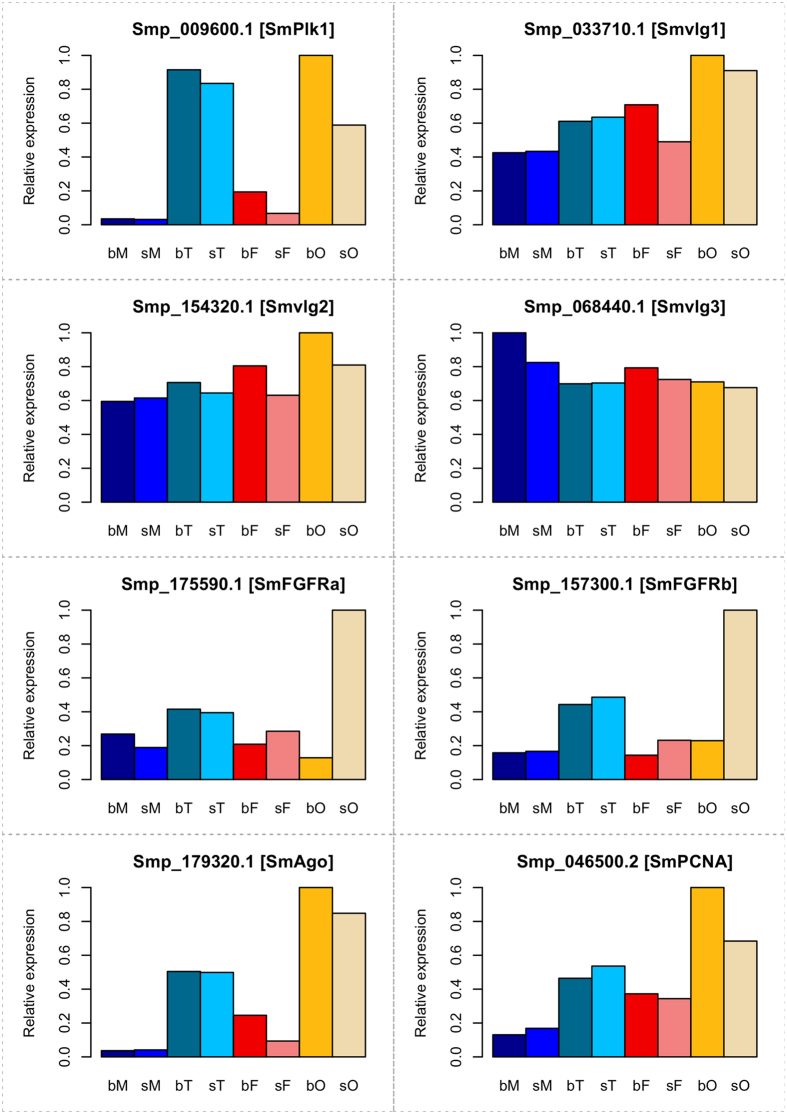
Transcript plots of exemplary genes involved in stem-cell proliferation and differentiation. Gene identifiers and Smp numbers are given. bM, bisex males; sM, single-sex males; bT, testes from bisex males; sT, testes from single-sex males; bF, bisex females; sF, single-sex females; bO, ovaries from bisex females; sO, ovaries from single-sex females. SmPlk1, polo-like kinase 1^24^, Smvlg1-2, vasa-like genes 1-3^25^, SmFGFRa/b, fibroblast growth factor receptor^20^,^26^, SmAgo, argonaute, and SmPCNA, proliferating cell nuclear antigen^23^.

From sporocyst-enriched genes analyzed in a different study, 581 were identified that shared similarities with planarian neoblast-enriched genes^23^, indicating their roles in stem-cell proliferation and differentiation. In our data sets these genes were transcribed at a higher level compared to the average transcript level of all genes. Particularly, this applied for their transcript levels in the gonads, in which 1.72–2.38 fold differences were determined (Supplementary Table S11). Among these genes, many have been shown in independent studies before to be important for gametogenesis like Polo-like kinase 1^24^, vasa-like genes 1–3^25^, FGFRs a and b^20^,^26^, or for neoblasts in planaria such as argonaute and PCNA^23^ (Fig. 4).

### Neural processes, a so far neglected aspect of schistosome male-female interaction and sexual development

**Figure 5:**
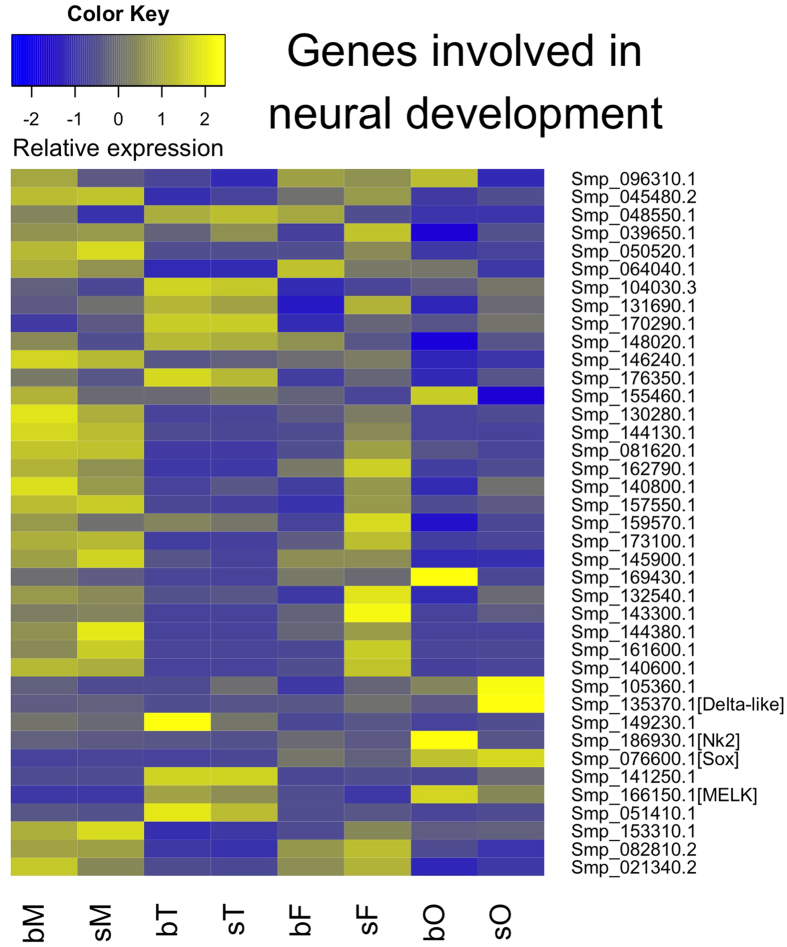
Hierarchical clustering of genes involved in neural processes. Blue and yellow colors indicate low and high transcript levels, respectively (see color key). Right labeling: Smp numbers of the 61 genes, whose function has been associated with neural processes^10^. Exemplary genes are indicated by name according to their annotation. Bottom line: all compared samples (bM, bisex males; sM, single-sex males; bT, testes from bisex males; sT, testes from single-sex males; bF, bisex females; sF, single-sex females; bO, ovaries from bisex females; sO, ovaries from single-sex females).

Considering data of a former study from our laboratory providing clear evidence for the involvement of neural processes during the male-female interaction of *S. mansoni*^19^, we compared the RNA-seq data to the previous ones obtained upon genome sequencing. Among the 61 identified genes with potential function in neural processes^10^, 39 were transcribed in the adult stage with varying transcript levels in whole worms or gonads (Fig. 5). Among these were MELK (Smp_166150), a proliferation-regulating serine/threonine kinase with functions in neural stem cells^27^, which was preferentially transcribed in testes and ovaries (Fig. 5; see also Fig. 2), the homeobox protein Nk2 (Smp_186930), a transcription factor involved in neural patterning^28^,^29^, which was preferentially transcribed in bO, the neurogenic delta-like protein (Smp_135370)^30^, which was found to be preferentially transcribed in sO, and the ovary-preferentially (bO, sO) transcribed transcription factor Sox (Smp_076600) (see also Fig. 2). The participation of Sox in developmental processes including the maintenance of embryonic and neural stem cells had been shown before^31^. The schistosome Sox ortholog was also identified as highly transcribed in the germ balls^32^, indicating a stem cell-associated function.

**Figure 6:**
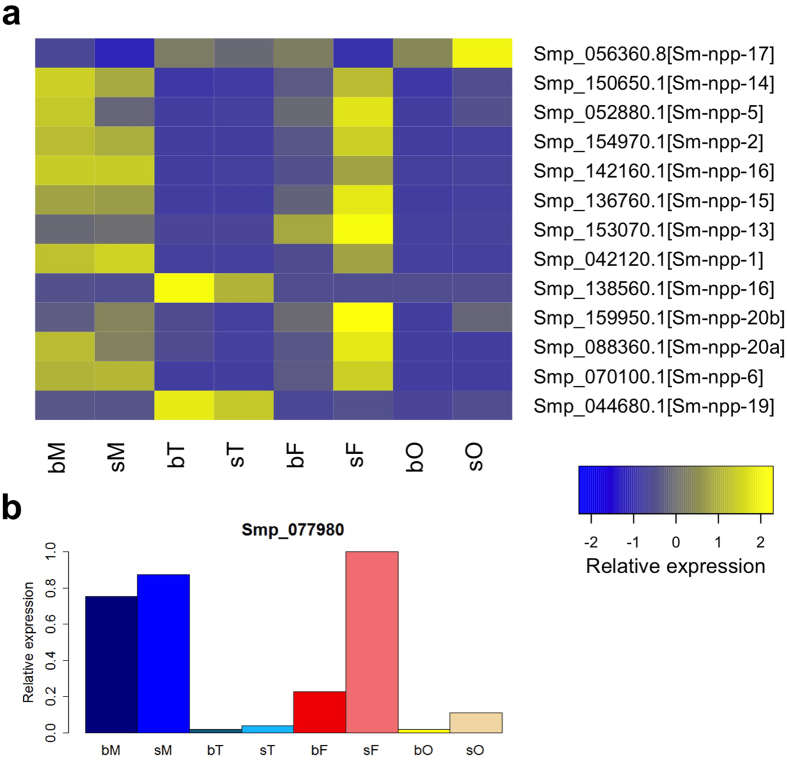
Expression of neuropeptide precursor and convertase genes. (**a**) Hierarchical clustering of neuropeptide precursor genes. Blue and yellow colors indicate low and high transcript levels, respectively (see color key). Right labeling: Smp numbers of the 13 neuropeptide precursor genes^35^. (**b**) Transcript profile of the neuropeptide convertase gene.

Neuropeptidergic signaling is known to play fundamental roles in flatworm locomotion, feeding, host-finding, regeneration and reproduction^33^. A recent study also highlighted the importance of neuropeptides in planarian germline development^34^. Furthermore, an *in silico* approach was performed to identify potential neuropeptides and precursors in flatworms^35^. Of these some occurred in our dataset being highly transcribed in gonads such as Sm-npp-19 (Smp_044680; Fig. 6a), which was found to be transcribed in all compared samples but transcripts values dominated in bT and sT. Sm-npp-17 (Smp_056360.8) transcript levels dominated in the gonads compared to whole worms with a clear pairing-influenced bias of sO > bO. Sm-npp-16 (Smp_138560) is probably transcribed testes-specifically with a bias of bT > sT. Transcript levels of Sm-npp-5 (Smp_052880) and Sm-npp14 (Smp_150650) dominated in adults with an interesting pairing-bias of bM > sM and sF > bF (Fig. 6). A gene encoding a NPF-like peptide (*npy*-8) was demonstrated before to be required for maintaining reproductive tissues in planaria^34^. According to BLAST analyses *S. mansoni* has no obvious NPY-like prohormone orthologs with high identity to those detected in *Schmidtea mediterrenea*^34^. Nonetheless, Sm-npp-20b (Smp_159950) is annotated as a neuropeptide Y ortholog and exhibits 57% identity at the amino acid level to NPY-8 from *S. mediterrenea*. The profile of Smp_159950 indicated low transcript amounts in all samples (Supplementary Table S12) and a comparably higher expression in adults with a pairing-bias of sM > bM and sF > bF. Thus it appears questionable whether Smp_159950 could be a NPY-8-like molecule fulfilling similar functions in schistosomes than the *S. mediterrenea* NPY-8. A similar finding was made for Sm-npp-20a which is represented by Smp_088360. Although it is annotated as a neuropeptide F ortholog, Smp_088360 exhibited 53% identity at the amino acid level to NPY-8 from *S. mediterrenea*. Again, low transcript amounts were found in all samples, but a comparably higher expression in adults with a pairing-bias of bM > sM and sF > bF. Finally, a prohormone convertase gene (Smp_077980) showed low abundance of transcripts in the gonads but a higher abundance in adults with similar levels in bM and sM as well as a female bias of sF > bF (Fig. 6b).

### Potential reference genes

A total of 15 candidate genes were found to be transcribed with ≤1.4-fold difference between any two samples. Thus, they may represent potential house-keeping genes for all samples analyzed and could serve as reference genes for quantitative analyses. These genes included syntaxin 6 (Smp_104960), ADP ribose pyrophosphatase (Smp_138760), and serine threonine protein phosphatase 2A (Smp_166290) (Supplementary Table S13).

### Overlap to microdissection data

In a previous study using laser microdissection to obtain reproductive tissues of schistosomes for RNA extraction and microarray analyses the authors found 4,450 genes enriched in the ovary and 2,171 genes enriched in the testis to be transcribed with a ≥2-fold difference^36^. Analyzing our data set the same way, we found 1,402 genes in the ovary and 2,108 genes in testis to be transcribed ≥2-fold compared to the whole worm controls (FDR < 0.005 for both; data not shown). These two data sets had 557 genes in common for the ovary and 677 genes for the testes (Supplementary Table S14). Furthermore, a correlation analysis was performed between the data of these two approaches using normalized intensity (microarray) and read counts (RNA-seq). Some variations were observed (Supplementary Fig. S8), which may be due to technical differences between the two different approaches applied.

## Discussion

RNA storage has frequently been observed in gonadal cells. In spermatozoa of eukaryotes stored mRNAs can be transported via sperm and sperm fluid into the oocyte where among others they contribute to epigenetic processes. In *Drosophila* and other organisms, maternal mRNAs are deposited in the oocyte and translated during the post-zygotic period to direct late stages of oogenesis and early stages of embryogenesis^37^. This provides an explanation for our finding that the majority of transcripts (≥73%) of whole *S. mansoni* found in our study were present in gonads. A similar result was obtained in a recent study with the liver fluke *Clonorchis sinensis*. Based on manual dissection of worm tissues the majority of genes of this hermaphroditic parasite were found to be transcribed in the ovary (72.4%) and testis (81.2%)^38^. Our analysis demonstrated a strong influence of pairing on transcript levels in the gonads, especially in the ovary. With respect to the unusual reproductive biology of schistosomes it was expected to find more DEGs in the ovary, which exceeded the number of DEGs in testes about 15 times. Among the genes with higher transcript abundance in bO are many involved in Ras-signaling, apoptosis and ribosome biogenesis, processes hypothesized before to be important for sexual maturation^14^,^39^,^40^,^41^.

Another interesting finding of the global data analysis was that the transcript profiles of sF were more similar to bM and sM than to bF. This pointed to an evolutionary background. In hermaphrodites including cestodes^42^, protandry is common as a form of sequential hermaphroditism. Here the development of the male gonad occurs ahead of that of the female which applies also to Spirorchidae, hermaphrodites parasitizing poikilotherms like turtles and the most closely related sister group of the *Schistosoma*tidae^43^,^44^. Evolution of dioecy in *Schistosoma*tidae may have advanced via androdioecy, in which hermaphrodites specialized in egg deposition in the vascular system, and larger adults developed further to functional males^43^. Subsequently, a “division-of-labor” between the genders represented the selection pressure for the evolution of the female gender to optimize reproductive success. This view is independently supported by recent studies indicating that sex allows faster rates of adaptation and the benefits of increasing genetic variation compensate for the short-term costs of sexual reproduction^45^. Indeed, in schistosomes physiological and metabolic functions are unequally distributed between male and female schistosomes when pairing has occurred^44^,^46^,^47^. Moreover, microarray studies showed that the diversity of genes transcribed in sF was larger than that of bF^48^. In this context transcript levels of genes with functions in egg production increased in bF. At the same time transcript levels of genes associated with motor functions decreased in bF, while transcript levels of these genes rose in bM compared to sM^48^. Also in our data set GO analysis showed higher transcript profiles of genes in bM that belong to the category “striated muscle contraction”. This suggests that the origin of dioecy in schistosomes resulted from the evolution of genes suppressing female function in males and, according to physiological and molecular data, also in sF, while male functions were suppressed in bF^43^. Thus the situation of schistosomes is similar to protandric hermaphrodites with respect to the direction of gonad development, testes first (independent of pairing) and the completion of ovary development second (only after pairing). This perspective is indirectly supported by the occurrence of pseudo-ovaries and vitellaria occasionally found in males in addition to testicular lobes^8^,^49^,^50^. The occurrence of female-gonad parts in males may represent an evolutionary set-back (atavism) and coincides with former results showing leaky transcription of female-specific genes associated with egg synthesis in males^19^. Thus we conclude that before pairing sF are more similar to males than to bF and with respect to the question of hermaphroditism versus dioecy, the complete functional separation of the sexes of schistosomes cannot be stated. Female schistosomes still need the constant pairing contact to complete sexual maturation, which represents an obvious reminiscence to their protandric ancestors and a transitional stage to complete dioecy.

Biological categories of specific interest included stem cell-associated genes, of which some such as the polo-like kinase SmPlk^24^ appeared to be preferentially transcribed in gonads (Fig. 4). The transcript profiles of others, such as vasa-like genes^25^, were heterogeneous in gonads and whole worms indicating additional roles in the neoblast-like cells that were recently detected in somatic tissues of *S. mansoni*^26^. Among the genes with testis- or ovary-specific and pairing-dependent transcription (categories 1.1 and 2.1), synaptotagmins were detected, sensory molecules responsible for Ca^2+^-dependent exocytosis and neurotransmitter release at synapses^51^,^52^. Corresponding to their proven role in spermatogenesis in mice^53^, planaria^54^ and *Macrostomum lignano*^55^, one *S. mansoni* ortholog of the RNA-binding protein ELAV was found as testis-specifically transcribed (category 1.2). For *Drosophila* but also higher eukaryotes ELAV proteins have been shown to be necessary for neural differentiation including the transition between stem cells and differentiation-committed cells and post-transcriptional processes such as cytoplasmic poly-adenylation^56^. This process elongates poly(A) tails after mRNA export to the cytoplasm and involves also other molecules such CPEBs. The schistosome CPEB1 ortholog (Smp_070360; category 2.1) was found to be specifically transcribed in bO, and in a former study CPEB1 transcripts were localized at the posterior part of the ovary of schistosome females^57^. In other organisms CPEBs fulfill roles during the cell cycle. They mediate the timely translation of stored mRNAs during oogenesis and early embryogenesis^22^,^58^,^59^. The 1,492.3-fold increase in the transcript level of CPEB1 upon pairing in the ovary clearly points to a specific and decisive role of this molecule for oocyte differentiation and early embryogenesis but also to post-transcriptional processes as control for pairing-induced processes during female sexual maturation in schistosomes.

MELK was initially identified as a maternally derived gene with functions in the unfertilized egg and pre-implantation embryo in mice^60^. Its transcript amount is elevated during mitosis because MELK phosphorylates the phosphatase Cdc25 to promote mitosis entry. Schistosome orthologs of MELK (Smp_166150; category 4.2) and Cdc25 (Smp_152200; category 1.1) were found to be transcriptionally active in testes although a pairing-dependent influence was only observed for Cdc25. Surprisingly, the latter was not transcribed in the ovary in contrast to MELK, which exhibited a pairing-dependent transcript profile in this organ (bO > sO). In *Caenorhabditis elegans, Danio rerio*, and *Xenopus laevis*, MELK controls cell division as well as the propagation and maintenance of organ-specific stem cells including neuroblasts^27^.

Remarkably, the obtained data included an unexpected high number of further DEGs putatively involved in neural processes such as the transcription factor Sox (Smp_076600) and genes with potential functions in neuropeptidergic signaling. Thus neural processes could play a role for gonad growth and differentiation in *S. mansoni*. This view is supported by first findings in planaria highlighting the role of NPY-8, a neuropeptide expressed in the nervous system of *S. mediterranea*, for reproduction^34^. In sexually mature planaria *npy-8* transcripts were found in dorsally located cells, including some cells associated with the testicular lobes, in cephalic ganglia, ventral nerve cords, the submuscular plexus, and pharyngeal cells but not inside the testicular lobes. Nevertheless, regressed testes without mature sperm were observed in *npy-8* knock-down (RNAi) animals. From these and other results the authors concluded that neuropeptides like NPY-8 may act in a neuroendocrine way to regulate reproductive development. On the other hand, peptides produced within the gonads may be involved in providing feedback signals to the CNS or other organs about the physiological status of the gonads, and/or they could serve as paracrine factors controlling germ cell maturation^34^. Our data correspond to such a scenario. Although the existence of a *npy-8* homolog in *S. mansoni* is still questionable, the amount of putative neuropeptides and their gonad- or whole worm-preferential/specific transcription patterns suggest not only the importance of neural processes for gonad differentiation processes but also the existence of endocrine and paracrine mechanisms influencing the reproductive biology of adult schistosomes. Moreover, some of these processes are obviously under the control of pairing which opens a new field of research aspects for the future.

Schistosomiasis and other platyhelminths-induced infectious diseases have a high medical and economic impact for humans and animals. Against this background it is alarming that no vaccines exist, and only a very limited repertoire of pharmaceuticals is available to fight parasitic flatworms, which justifies the fear of upcoming resistance. Thus identifying novel strategies to fight these parasites has high priority. In this context, targeting reproductive function represents a promising route because life cycles and also the egg-associated pathologic consequence of infection could be interrupted, as it is the case for schistosomiasis. Not until applying the novel protocol for organ isolation as well as using organ RNA of gonads and adults of different pairing-experience for RNA-seq, we were able to generate comprehensive gene transcription catalogs combined with functional aspects regarding the reproductive biology of *S. mansoni*. Here especially the stem cell-associated but also the neural processes are striking and may represent viable targets for novel treatment options including the eradication of schistosomes and perhaps also other parasitic flatworms.

## Methods

### Ethic statement

Animal experiments were in accordance with the European Convention for the Protection of Vertebrate Animals used for experimental and other scientific purposes (ETS No 123; revised Appendix A) and have been approved by the Regional Council (Regierungspraesidium) Giessen (V54-19 c 20/15 c GI 18/10).

### Schistosome maintenance

For maintaining the life cycle, *Biomphalaria glabrata* snails were used as intermediate hosts and Syrian hamsters (*Mesocricetus auratus*) as final hosts. The strain of *S. mansoni* originated from a Liberian isolate obtained from Bayer AG (Monheim, Germany). Bisex (=mixed sex) and single-sex (=unisexual) worm populations were generated by polymiracidial and monomiracidial intermediate-host infections, respectively^61^. Adult worms were obtained by hepatoportal perfusion 46 (bisex) or 67 (single-sex) days post infection (p.i.) and transferred to Petri dishes containing M199 medium (Sigma-Aldrich; supplemented with 10% newborn calf serum (NCS), 1% HEPES [1 M] and 1% ABAM-solution [10,000 units penicillin, 10 mg streptomycin and 25 mg amphotericin B per ml]) in groups of at most either 20 couples, or 25 males, or 25 females per Petri dish (6 cm diameter) as described elsewhere^17^. If required, couples from bisex infections were manually separated by pipetting or with feather-weight tweezers to ensure the paired status of a female or a male for further processing such as gonad isolation or RNA extraction.

### Ovary and testes isolation

Gonad isolation was performed by a detergent- and enzyme-based isolation procedure^17^. In short, worm couples were separated immediately after perfusion, and about 50 (males and bisex females) or 100 (single-sex females) individuals were transferred into 2 ml Eppendorf vessels, respectively, and washed with 2 ml M199 (non-supplemented) medium. Tegument removal was achieved by incubating worms for 2 × 5 min (females) or 3 × 5 min (males) in 400 μl stripping buffer, respectively (0.1% of each: Brij 35 (Roth), Nonidet P40 (NP-40, Fluka), Tween 80 (Sigma), and Triton X-405 (Sigma) in DEPC-PBS, pH 7.2–7.4; 0.2 μm filtered before use) at 1,200 rpm and 37 °C on a thermal shaker (Biosan TS-100, Latvian Republic). Afterwards, the worms were washed with 2 ml M199 (non-supplemented, 3x), the medium removed, and enzymatic digestion started by incubating each worm sample with 300–500 μl elastase (Sigma E0258; 5 U/ml in non-supplemented M199) for 20–40 min at 650 rpm and 37 °C. The process of tissue digestion was checked microscopically (Leica). The tube contents were transferred to 2.5 cm Petri dishes filled with M199 (2 ml each). Intact gonads were collected by pipetting with 10 μl tips, and transferred 2–3 times to new dishes for purification. Organs collected this way were transferred into RNase-/DNAse-free vessels on ice, centrifuged at 6,000 *g* for 2 min and frozen at −80 °C after removing the supernatant. In parallel, 5–10 organs were stained 10 μl Trypan Blue (0.4%; Sigma) for vitality check with similar results as described before^17^. Phase-contrast images of the gonads were taken on an Olympus IX 81 microscope.

### RNA extraction

Total RNA was extracted with Trizol/Chloroform as previously described^17^. Male or female worms (20 each) were collected immediately after perfusion into separate 1.5 ml vessels, before 300 μl PeqGOLD TriFast reagent (Peqlab) was added to each tube and the worms mechanically homogenized with plastic pistons. After filling the reagent to 500 μl, the vessels were kept at room temperature (RT) for 5 min. For cleaning, 100 μl chloroform was added to each vessel followed by vigorous shaking for 15 sec. After settling down for 3–10 min, centrifugation was performed at 12,000 *g* and 4 °C for 10 min. Each aqueous phase was transferred to new tubes, and 250 μl of isopropanol added for RNA precipitation at −20 °C overnight (o/n). Afterwards, the tubes were centrifuged for 10 min at 12,000 *g* and 4 °C. The supernatant was removed, ice-cold 75% ethanol (1 ml) added to each pellet and centrifugation was repeated before the pellets were washed again, dried at RT for 5 min, and resuspended in 50 μl DEPC-dH_2_O each.

Total RNA from gonads (50–300 each) was extracted by adding 50 μl TriFast-solution to each tube immediately after removing the medium. To completely disrupt gonad cells, three freeze-thaw cycles were performed in liquid nitrogen. RNA was precipitated at −20 °C o/n by adding 250 μl isopropanol and glycogen (0.07 mg/ml; RNase-free PeqGOLD glycogen, Peqlab). After precipitation, the RNA was concentrated by centrifugation at 4 °C and 12,000 *g* for 10 min. The supernatant was removed and the RNA pellet washed with 75% ice-cold ethanol (500 μl), dried at RT, and finally resuspended in 20 μl DEPC-H_2_O.

Quality and quantity of total RNA from whole worms and gonads were checked by electropherogram analysis (Agilent 2100 Bioanalyzer; Agilent RNA 6000 Pico Kit) according to the manufacturer’s instruction (Agilent Technologies).

### RNA-seq analyses and data processing

From each sample 100 ng of total RNA was used for RNA-seq. In total eight (1–8) different samples were generated and analyzed. These covered males (M) that were paired and obtained from bisex infections (1: bM) or males without pairing experience obtained from single-sex infections (2: sM), testes obtained from bM (3: bT) or sM (4: sT), females (F) that were paired and obtained from bisex infections (5: bF) or females without pairing experience obtained from single-sex infections (6: sF), and ovaries obtained from bF (7: bO) or sF (8: sO).

Three biological replicates (*n* = 3) were generated for each sample, except sO (*n* = 2), which are extremely difficult to obtain in high numbers due to the low growth rate of single-sex female schistosomes within final hosts^62^ and the low amount of total RNA in the immature ovary^17^. For worm samples, each replicate (50 sF worms/replicate and 10 worms/replicate for all other samples) was obtained from a single hamster. For gonad samples, each replicate (containing 54–813 organs) was obtained from worms harvested from several hamsters.

Sample libraries (7 × 3 + 1 × 2 = 23 in total) were made with TruSeq RNA Library Preparation Kit (Illumina). All libraries were multiplexed in one pool (23-plex tags) and sequenced on Illumina HiSeq 2500 with 100 bp paired-end reads. This represents >40 Gb data in total, which equals 1.74 Gb data per sample. This corresponds to approximately 100x coverage of ~16 Mb *S. mansoni* transcriptome^63^. Each sample was sequenced in parallel in two lanes (=two technical replicates), and afterwards the mapping sequence files (.bam) were merged. Samples were named consecutively with replicate numbers, for instance, bM1, bM2 and bM3 for the three independent bM replicates.

Data processing was performed by TopHat (v2.0.8b; command: tophat -g 1 –library-type fr-firststrand -r 200–mate-std-dev 100 -a 6 -i 10 -I 40000 –microexon-search –min-segment-intron 10 –max-segment-intron 40000) for mapping, followed by HTSeq (v0.5.4; parameters: -f bam –s reverse) for counting reads, edgeR (v3.6.7) for differential gene expression analysis and Blast/Gene Ontology (GO)/KEGG pathway mapping. Mapping sequence files were merged by SAMtools “merge” (http://www.htslib.org/doc/samtools.html). Afterwards, counts were calculated by “htseq-count” from the merged “bam” files (23 in total, one per sample). For protein-coding genes/transcripts with different splice variants, only the longest were recorded. All sequence data can be obtained from the European Nucleotide Archive (ENA) under the study accession number ERP016356, and from ArrayExpress under the accession number E-ERAD-516.

To identify differentially transcribed genes, the R package edgeR was used with read counts as input^64^ and Pearson’s correlations between replicates were calculated. In following analysis three expression measurements were used. (1) Raw RPKM (reads per kilobase per million mapped reads) values of each gene across all samples were calculated to unravel the top 100 highly transcribed genes and to filter low abundantly transcribed tags. For the latter purpose we only selected genes whose raw RPKM was >2 in all replicates of at least one sample. To assess the arbitrary effect, numbers of genes under other RPKM thresholds (0–10) were also calculated; (2) Raw read counts after RPKM filtration were imported to edgeR for normalization using and the TMM method by the function *calcNormFactors*, followed by the differential expression analysis; (3) RPKM values based on normalized reads were calculated using the function *rpkm* in edgeR with the parameter ‘normalized.lib.sizes = TRUE’, and the mean RPKMs of replicates were used for illustrative purposes (e.g. barplots and heatmaps), where the R package gplots (v2.16.0)^65^ was used.

To assess overall similarities and to measure the distances among samples, three different approaches were applied. First, multidimensional scaling analysis was performed in edgeR according to Biological Coefficient of Variation (BCV) with default parameters. Second, the sample distance matrix was generated using the R package DESeq2 (v1.4.5) based on *regularized-logarithm transformation*^66^. Third, to get an overview of gene transcription patterns and to facilitate the understanding of sample relations, a hierarchical clustering was achieved based on mean RPKM values of all genes.

To investigate the effects of pairing, gender, or tissue on gene transcription, a GLM (generalized linear model) approach^67^ with defined contrasts was applied to calculate log_2_FC (log-2 fold change) and FDR (false discovery rate) values. The comparisons were set as follows: (i) pairing effect: bM/sM (bM versus sM), bT/sT, bF/sF, bO/sO; (ii) gender effect: bM/bF, sM/sF, bT/bO, sT/sO; (iii) tissue effect: bT/bM, sT/sM, bO/bF, sO/sF. The cut-offs for selecting significantly differentially expressed genes (DEGs) were: fold-difference >1.5 for all comparisons; FDR < 0.05 for bM/sM and bT/sT, FDR < 0.005 for the rest. Volcano plots were generated based on log_2_FC and log_10_FDR values for each comparison.

To compare the pairing effect on levels of transcripts in testes (=bT/sT) and ovaries (=bO/sO), log_2_FC and FDR values originating from these two data sets were combined for individual genes. In addition, DEG analyses also included sF versus sM, and bF versus bM, which aimed to assess the differential relationship of sF and bF to the males. Corresponding heat map was made based on the log_2_FC values of bM/bF comparison.

Candidates for genes with house-keeping functions were discovered by using the GLM approach in edgeR, which works as an ANOVA-like test to find genes whose transcription did not show significant differences between any two of the eight samples^67^. The cut-off used here was 5% FDR and 1.4-fold difference. Gonad-enriched transcripts were discovered by comparing their average expression in gonad samples to those in worm samples and setting the cutoff at 5-fold. Annotations were obtained from the GeneDB database v 5.0 (http://www.genedb.org). Conserved domains were identified by NCBI Batch CD-Search on 20 February 2015 using the default database and parameter (cdd v3.13, E-value cut-off 0.01). Orthologs of *S. mansoni* proteins to the Kyoto Encyclopedia of Genes and Genomes (KEGG) database were obtained from the KEGG Automatic Annotation Server (KAAS; http://www.gemone.jp/tools/kaas/) in February 2015 using the BBH method. Subsequently, KEEG pathway mapping^68^ was performed using KEEG Mapper (http://www.genome.jp/kegg/tool/ map_pathway.html). Gene ontology^69^ (GO) enrichment analysis was performed with the Bioconductor topGO package (v2.16.0) for R^70^, where *p* < 0.01 was set for identifying significantly enriched categories.

## Additional Information

**How to cite this article**: Lu, Z. *et al*. Schistosome sex matters: a deep view into gonad-specific and pairing-dependent transcriptomes reveals a complex gender interplay. *Sci. Rep.* **6**, 31150; doi: 10.1038/srep31150 (2016).

## Supplementary Material

Supplementary Information

Supplementary Information

Supplementary Information

Supplementary Information

Supplementary Information

Supplementary Information

Supplementary Information

Supplementary Information

## Figures and Tables

**Table 1:** Numbers of differentially transcribed genes.

Comp.	Pairing				Gender				Tissue			
	bM/sM	bF/sF	bT/sT	bO/sO	bM/bF	bT/bO	sM/sF	sT/sO	bT/bM	bO/bF	sT/sM	sO/sF
#Up	313	1,591	96	1,752	2,444	2,458	680	1,800	2,741	2,209	2,792	2,134
#Down	113	2,157	147	1,848	1,939	2,171	765	1,037	2,737	2,325	2,617	2,946
#Total	426	3,748	243	3,600	4,383	4,629	1,445	2,837	5,478	4,534	5,409	5,080
%DEGs	5.1	43.7	3.1	45.4	51.5	58.1	16.8	34.3	62.4	51.5	68.2	57.5

Numbers were obtained by setting FDR < 0.05 (for bM/sM and bT/sT) or FDR < 0.005 (for all the other comparisons) and fold-difference ≥1.5. DEGs: differentially expressed genes.
