# Supplementary material for: Schistosome sex matters: a deep view into gonad-specific and pairing-dependent transcriptomes reveals a complex gender interplay
Source: Sci Rep. 2016 Aug 8;6:31150. doi: 10.1038/srep31150 (PMC4976352; doi:10.1038/srep31150)
Supplement: Supplementary Information [file srep31150-s1.doc]

**Schistosome sex matters: a deep view into gonad-specific and pairing-dependent transcriptomes reveals a complex gender interplay**

Zhigang Lu^1^, Florian Sessler^2^, Nancy Holroyd^2^, Steffen Hahnel^1^, Thomas Quack^1^, Matthew Berriman^2^, Christoph G. Grevelding^1*^

**Affiliations:**

^1^BFS, Institute of Parasitology, Justus-Liebig-University, Giessen, Germany.

^2^Wellcome Trust Sanger Institute, Wellcome Genome Campus, Hinxton, United Kingdom.

*Correspondence to: Christoph G. Grevelding ([Christoph.Grevelding@vetmed.uni-giessen.de](mailto:Christoph.Grevelding@vetmed.uni-giessen.de)).

Supplementary Information

**Supplementary Notes**

Top highly transcribed genes among all worm and gonad samples

Based on their average RPKMs in all 23 samples, the top 100 highly transcribed genes were selected for hierarchical clustering (Supplementary Fig. S2; Supplementary Table S4). Among those investigated, the gene p14 (Smp_131110), encoding an egg-shell precursor protein, is one of the most abundantly expressed genes in schistosomes^1,2^, and showed the highest level of transcription in our dataset (average RPKM = 17275.6). Several ubiquitously and abundantly transcribed genes were also listed, including those encoding GAPDH (Smp_056970), HSP70 (Smp_106930) and α-tubulin (Smp_090120). Beside these, there were transcripts encoding 35 different ribosomal proteins. Different gene transcription patterns were detected within these clusters. For example, in the testes of both male samples, highest transcription was seen for genes encoding histone H3 (Smp_082240), histone H1 (Smp_003770) and α-tubulin (Smp_090120). Abundantly transcribed in bO were genes encoding among others translation elongation factors and ribosomal proteins.

DEG categories

DEGs (differentially expressed/transcribed genes) found in this work were divided into 8 categories (1.1 - 4.2) according to their testis/ovary-dependent/independent or pairing-dependent/ independent transcription profiles (Supplementary Fig. S8). Here these categories are listed and two representative examples of differential gene transcription are shown for each category (Fig. 2 and Supplementary Fig. S4). The complete lists of genes in each category are given in Supplementary Table S11.

Category 1.1: Testis-preferential/specific and pairing-affected genes. A total of 42 genes were found to be preferentially (or specifically) transcribed in testis and to be significantly affected by paring. Among these, transcript levels of 39 genes increased after pairing. This category included *cdc25* (Smp_152200), a gene encoding a cell division cycle phosphatase, whose transcript amount was elevated 1.75-fold after pairing. Furthermore, a 2-fold increase in the transcript level was determined for a *synaptotagmin* gene (Smp_150920), which codes for a Ca^2+^-sensing transmembrane protein.

Category 1.2: Testis-preferential/specific and pairing-unaffected genes. A total of 436 genes showed no significance in their transcript fold-changes following pairing in testes. This category included the RNA-binding protein gene *elav* (embryonic lethal, abnormal visual system; Smp_194950) and the transcription factor *spatial* (stromal protein associated with thymii and lymph node; Smp_194750), whose mouse ortholog is involved in spermatogenesis^3^.

Category 2.1: Ovary-preferential/specific and pairing-affected genes. A total of 309 genes were found to be preferentially transcribed in ovaries and significantly affected by pairing. In this category a *cpeb* (cytoplasmic polyadenylation element binding) gene (Smp_070360) with potential function in post-zygotic translational control^4^ and a *synaptotagmin XIV* gene (Smp_150350) were detected. Their transcript levels were pairing-dependently elevated in female ovaries 1,492.3-fold and 65.8-fold, respectively.

Category 2.2: Ovary-preferential and pairing-unaffected genes. The occurrence of transcripts of 171 genes appeared ovary-preferential (or specific) and not significantly affected by pairing. Among these was a SOX transcription factor (Smp_076600), which can have diverse functions including gonadal and neural processes^5^, as well as a regulator of G-protein signaling (Smp_210800) were found.

Category 3.1: Pairing-affected genes in both gonads. A total of 139 genes appeared to be significantly differential transcribed in both testes and ovaries upon pairing. In this category a potential potassium channel of the TWIK (tandem of P domains in a weakly inward rectifying K⁺ channel; Smp_147550) family was found, whose transcript increased upon pairing in testes 2.3-fold and in ovaries 34.7-fold. Furthermore, the micro exon gene MEG 13 (Smp_127990) belonged to this category^6^, whose transcript level decreased upon pairing 2.2- or 6.8-fold in testes or ovaries, respectively.

Category 3.2: Pairing-unaffected genes in both gonads. Transcript profiles of 4,100 genes did not show significant changes by pairing in both gonads. Among these was *lin 9* (abnormal cell lineage; Smp_133660), a gene with potential function in cell-cycle regulation and early embryogenesis^7^ and  a leucin zipper protein (Smp_124570). Furthermore, the vasa-like genes 1-3 ^8^ (Smp_033710, Smp_154320 and Smp_068440) and protein tyrosine kinases (PTKs) such as SmTK3 (Smp_212730/Smp_151300), SmTK5 (Smp_136300) and VKR2 (153500) were found here. These PTKs have been shown before to be transcribed in the gonads of *S. mansoni* and to be involved in reproduction-associated differentiation processes^9–11^.

Category 4.1: Transcription of genes affected by pairing in testes but not in ovaries. The transcript amounts of 62 genes were significantly affected by pairing in testes but not in ovaries. This included a gene that is predicted to encode a von-Willebrand factor A (vWA) domain-containing protein (Smp_127480) and a putative DNA double-strand break repair rad50ATPase (Smp_181450).

Category 4.2: Transcription of genes affected by pairing in ovaries but not in testes. The transcription amounts of 3,152 genes were found to be significantly affected by pairing in ovaries but not in testes. Among these was MELK (maternal embryonic leucine zipper kinase; Smp_166150), a kinase with potential stem-cell function^12^, whose transcript level was elevated 1.9-fold in the ovary after pairing. As an example the gene encoding a HMG-CoA (3-hydroxy-3-methylglutaryl-coenzyme A) synthase (Smp_198690) was also shown.

Furthermore, PTKs such as SmTK4 (Smp_149460), SmTK6 (Smp_006920), VKR1 (Smp_019790), and FGFRa/b (Smp_175590 and Smp_157300) were found, which are also known to be transcribed in the gonads of *S. mansoni*^9,13,11,14^.

**Supplementary Figures**


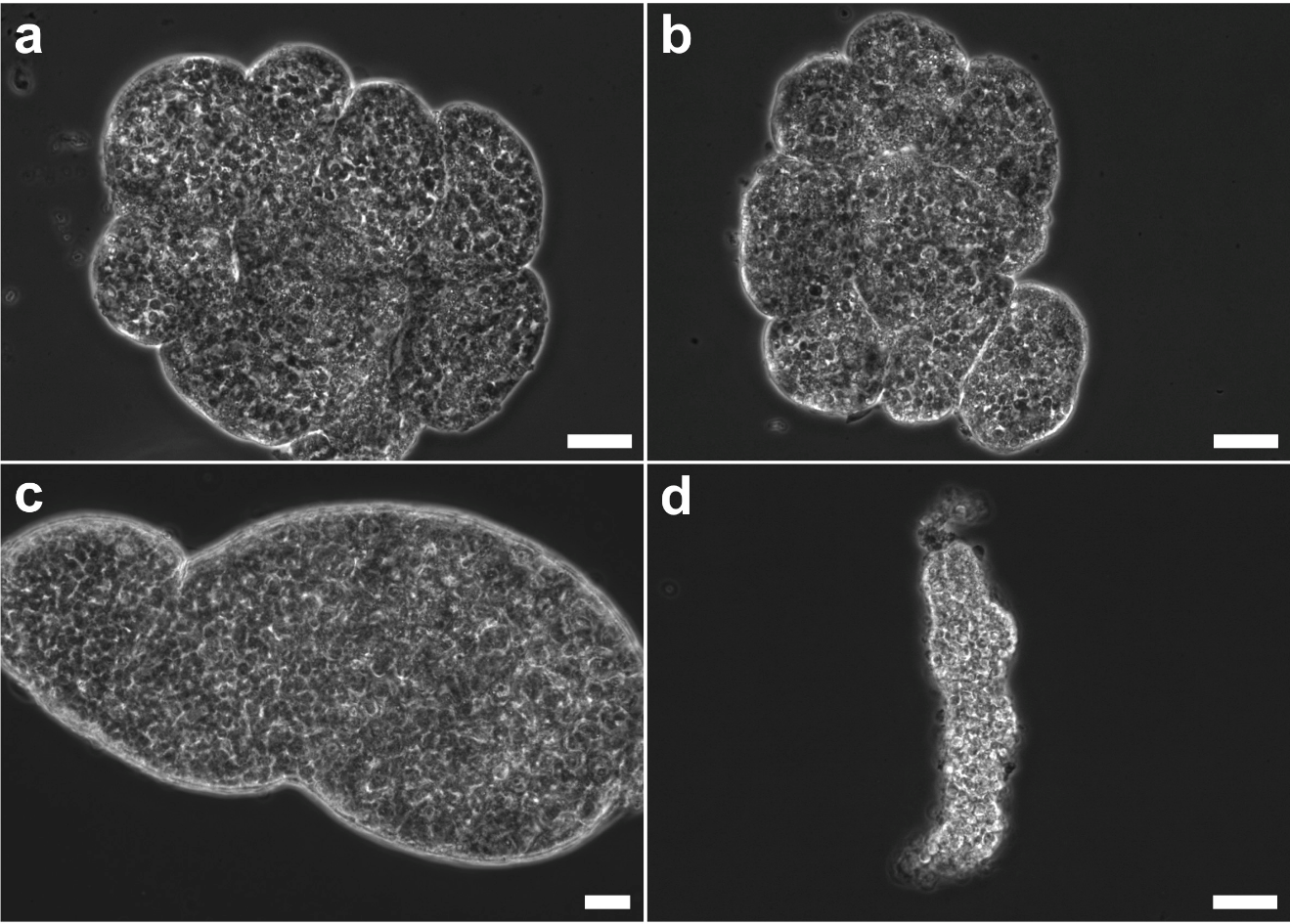


**Supplementary Figure 1. Microscopic analyses of isolated gonads from *S. mansoni*.** Phase contrast microscopy showing testes (**a**, **b**) and ovaries (**c**, **d**) from paired (**a**, **c**) and unpaired (**b**, **d**) adult worms obtained by the organ-isolation procedure^15^. Similar to previous findings^16^ testes of paired (bT) or unpaired (sT) males, obtained by single-sex or bisex infections, respectively, exhibited comparable sizes (155.0 **±** 5.5 µm in length) and consisted of 8-10 individual lobes containing spermatocytes at different stages of development. In contrast, the size of ovaries from paired females (bO; 236.7 **±** 15.3 µm in length) considerably exceeded that of unpaired females (sO; 116.0 **±** 0.7 µm in length), whose ovaries contained only undifferentiated oogonia. The shape of the sO was bean-like, whereas the bO appeared bulb-like and consisted of two parts: a smaller anterior part containing oogonia, and a larger posterior part containing mature, primary oocytes. Scale bars: 20 µm.

**
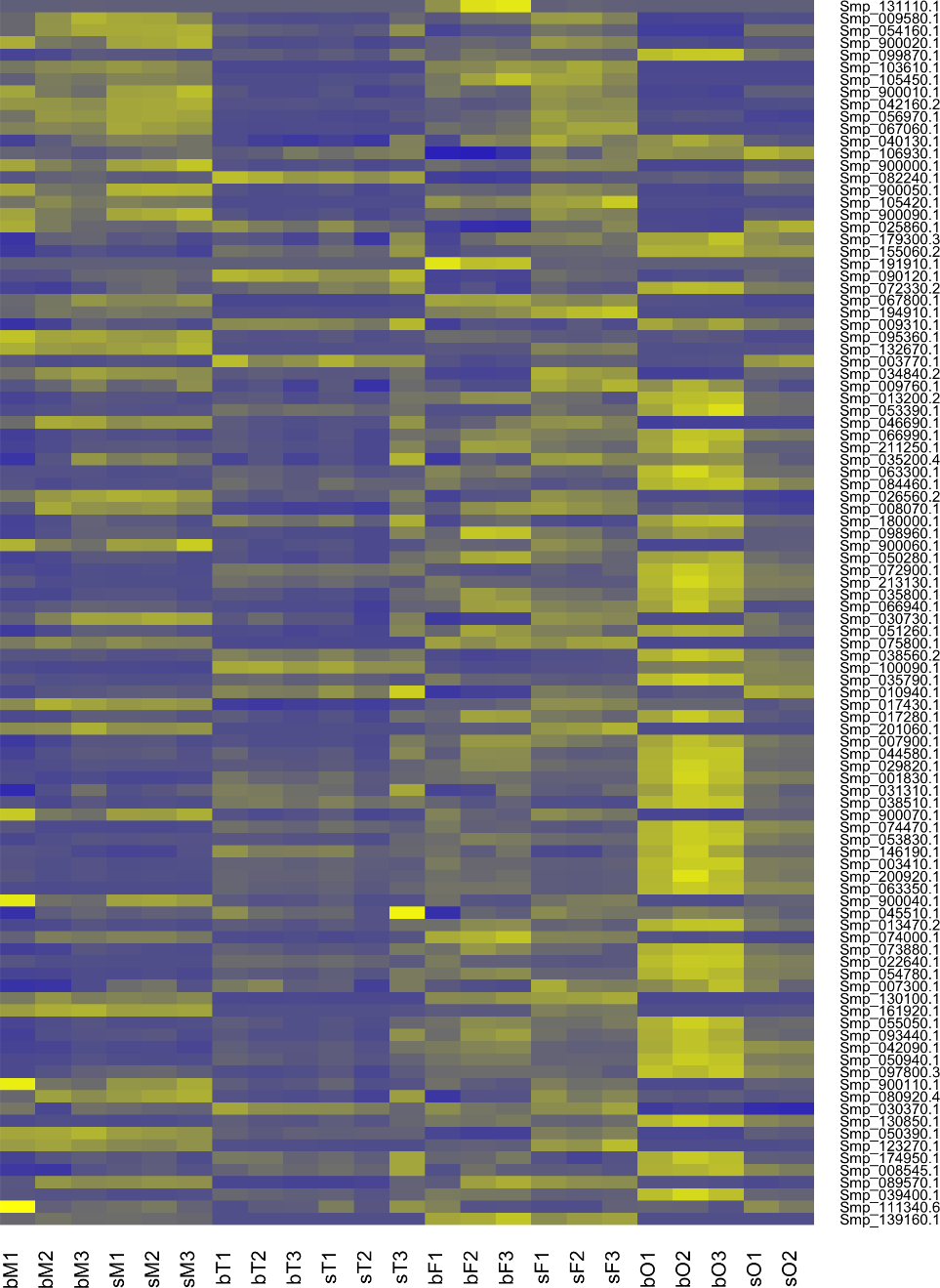
**

**Supplementary Figure 2. Hierarchical clustering of the top 100 highly transcribed genes.** Blue and yellow colors indicate lowest and highest transcript levels among samples, respectively. Right labeling: Smp numbers of the 100 genes in a descending manner from top to bottom according to their RPKM values. Bottom line: all samples (bM, bisex males; sM, single-sex males; bT, testes from bisex males; sT, testes from single-sex males; bF, bisex females; sF, single-sex females; bO, ovaries from bisex females; sO, ovaries from single-sex females) including the biological replicates (indicated by 1, 2 or 3).


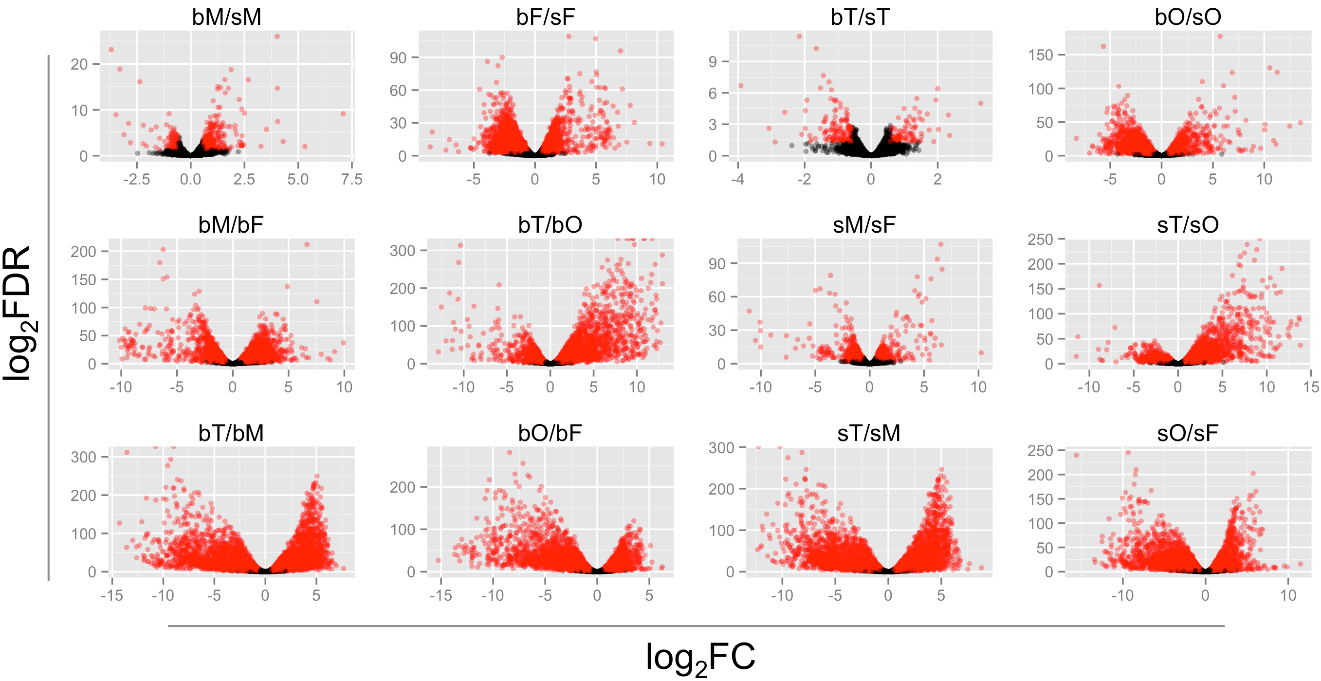
 Supplementary Figure 3. Volcano plots revealed differential gene transcription in various comparisons. Red points indicated DEGs (>1.5-fold difference; FDR < 0.05 for bM/sM and bT/sT; FDR < 0.005 for the rest), and black points indicated non-DEGs. Upper panel, pairing effect: transcripts of paired samples compared to their pairing-unexperienced counterparts. Middle panel, gender effect: male samples compared to female samples. Lower panel, tissue effect: gonad samples compared to their worm counterparts.

**
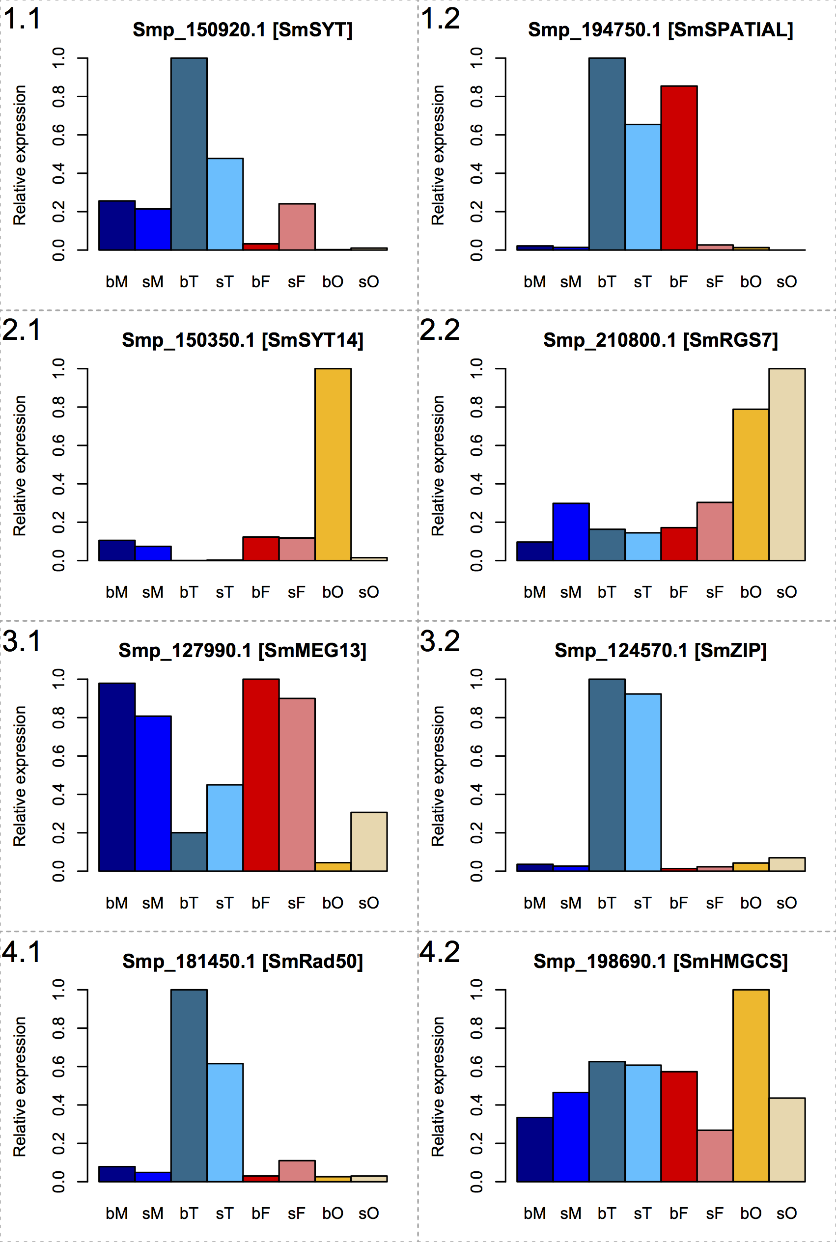
**

**Supplementary Figure 4. Exemplary genes from the eight categories (Cat. 1.1 - 4.2) and their transcript profiles on the basis of relative expression.** To provide more information about the transcript profiles of categorized genes, we added their relative expression also for the adult samples.

Cat. 1.1-1.2: Smp_150920 is a putative synaptotagmin gene and Smp_194750 is a putative spatial (stromal protein associated with thymii and lymph node) gene; Cat. 2.1-2.2 Smp_150350 is a gene coding for another synaptotagmin, synaptotagmin XIV, and Smp_210800 codes for a regulator of G protein signaling (RGS domain). Cat. 3.1-3.2: Smp_127990 represents a micro-exon gene (MEG) 13 and Smp_124570 represents a leucin zipper protein-coding gene. Cat. 4.1-4.2: Smp_181450 encodes a DNA double-strand break repair rad50ATPase and Smp_198690 encodes a HMG-CoA (3-hydroxy-3-methylglutaryl-coenzyme A) synthase.

**
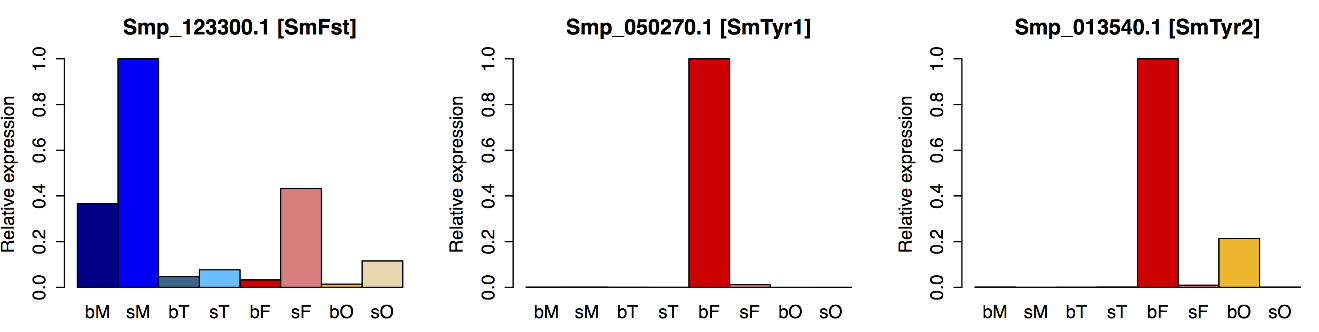
**

**Supplementary Figure 5. Exemplary transcript profiles.** Transcript profiles of the *S. mansoni* genes follistatin (SmFst), tyrosinase 1 (SmTyr1) and tyrosinase 2 (SmTyr2); bM, bisex males; sM, single-sex males; bT, testes from bisex males; sT, testes from single-sex males; bF, bisex females; sF, single-sex females; bO, ovaries from bisex females; sO, ovaries from single-sex females).


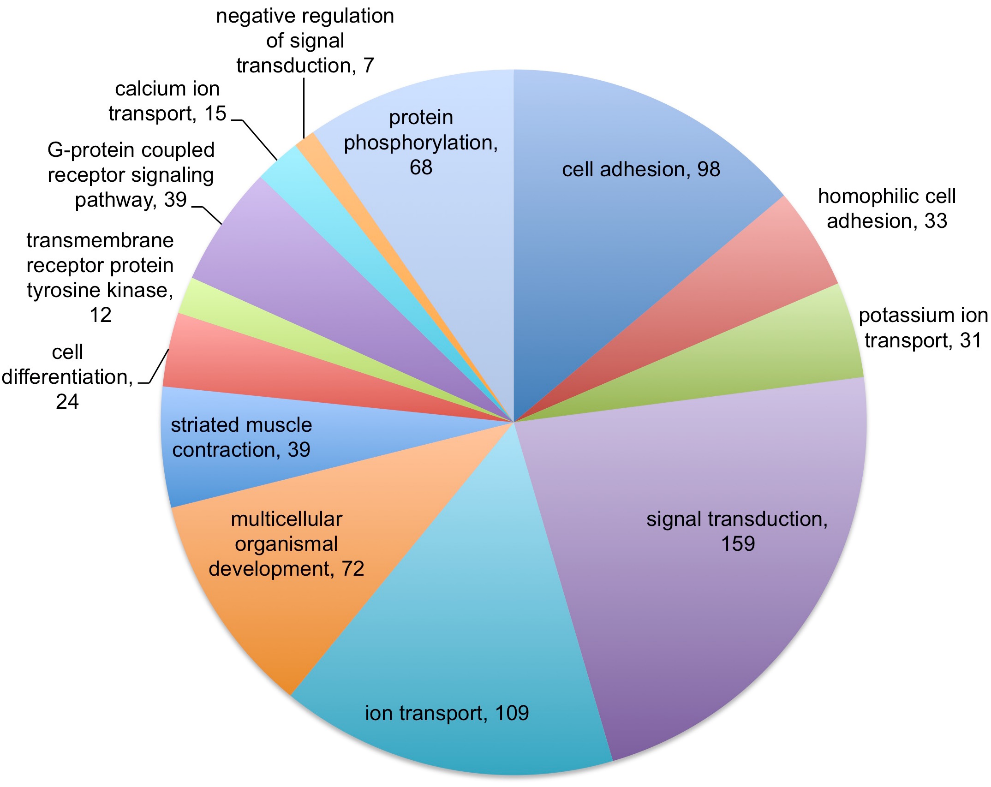


Supplementary Figure 6. Gene ontology enrichment of gene transcripts that were found to be less abundant in bF than in sF, and that were not differentially transcribed between sF and sM. The main biological processes they are involved in include signal transduction, ion transport, and cell adhesion.

**
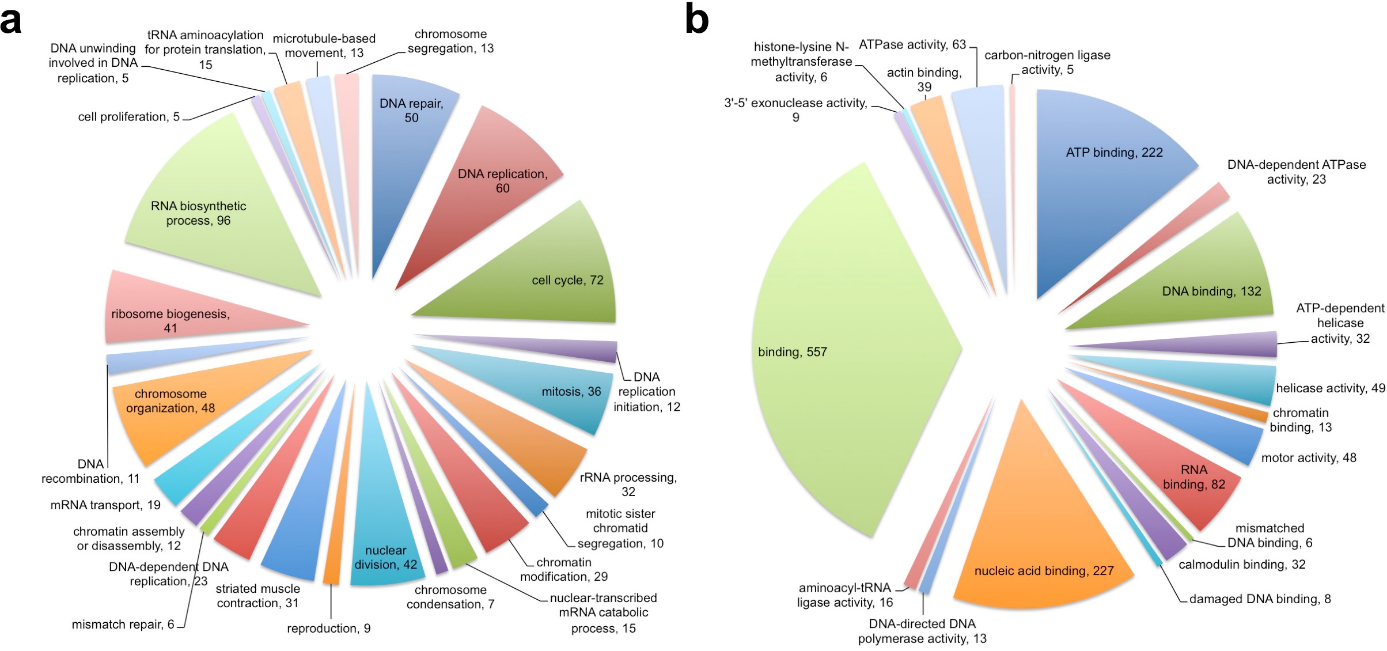
**

**Supplementary Figure 7. Gene ontology analysis.** Enrichment was done for genes whose transcripts occurred more abundantly in the gonads than in whole worms by biological process (**a**) and molecular function (**b**).

**
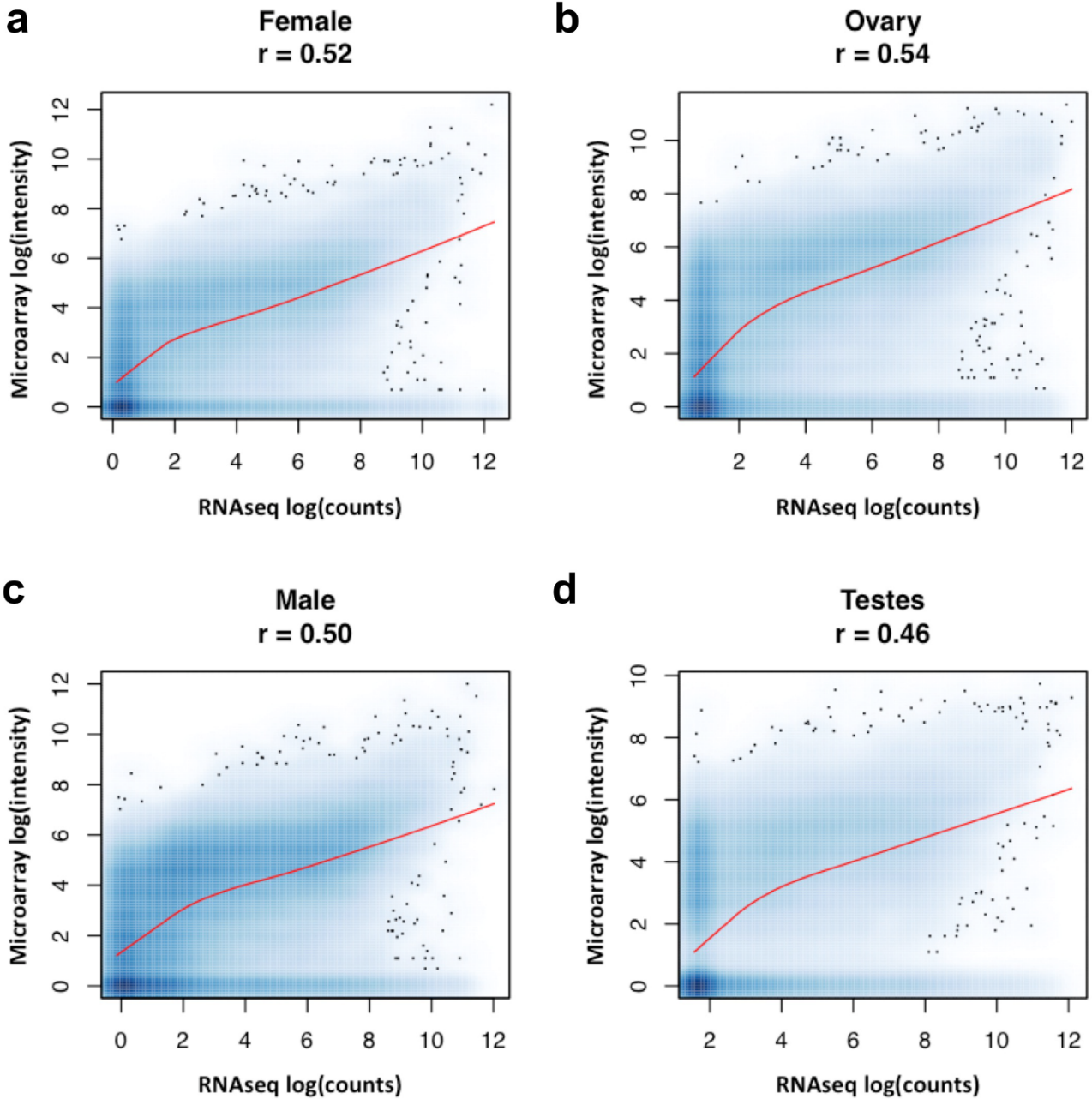
**

**Supplementary Figure 8. Correlations between RNA-seq data and microarray data.** Plotting RNA-seq counts against normalized intensity values of the microarray data sets. Local regression line is plotted in red. Correlations were calculated between whole paired female samples (bF; **a**), ovaries from paired females (bO; **b**), paired males (bM; **c**), and testes from paired males (bT; **d**).

**Supplementary Table S1.**

RNA-seq reads statistics

| **Sample** | **Total** | **Mapped** | **Prop.**  **paired** | **Singletons** | **Unmapped** | **%unmapped** |
| --- | --- | --- | --- | --- | --- | --- |
| bM1 | 82,157,621 | 71,801,485 | 57,615,006 | 5,011,317 | 10,356,136 | 12.61 |
| bM2 | 61,413,861 | 51,649,806 | 42,911,100 | 2,509,444 | 9,764,055 | 15.90 |
| bM3 | 58,786,566 | 51,179,327 | 42,452,604 | 2,580,515 | 7,607,239 | 12.94 |
| sM1 | 63,319,818 | 53,852,252 | 45,111,588 | 2,701,856 | 9,467,566 | 14.95 |
| sM2 | 63,888,404 | 53,738,964 | 44,763,160 | 2,691,940 | 10,149,440 | 15.89 |
| sM3 | 54,258,554 | 47,564,797 | 38,200,040 | 2,435,665 | 6,693,757 | 12.34 |
| bT1 | 43,373,743 | 38,119,993 | 30,926,682 | 2,511,925 | 5,253,750 | 12.11 |
| bT2 | 43,840,647 | 32,048,217 | 26,063,698 | 2,199,055 | 11,792,430 | 26.90 |
| bT3 | 65,099,133 | 48,212,772 | 24,158,538 | 2,332,190 | 16,886,361 | 25.94 |
| sT1 | 44,595,566 | 38,876,508 | 29,982,900 | 2,376,020 | 5,719,058 | 12.82 |
| sT2 | 56,767,832 | 49,722,004 | 40,360,354 | 3,168,100 | 7,045,828 | 12.41 |
| sT3 | 68,875,916 | 56,457,897 | 46,779,174 | 2,740,915 | 12,418,019 | 18.03 |
| bF1 | 42,049,886 | 36,204,716 | 19,131,978 | 2,683,370 | 5,845,170 | 13.90 |
| bF2 | 56,720,483 | 45,482,737 | 33,138,056 | 4,739,835 | 11,237,746 | 19.81 |
| bF3 | 48,461,371 | 39,592,784 | 28,397,394 | 4,281,282 | 8,868,587 | 18.30 |
| sF1 | 62,724,794 | 54,360,068 | 45,920,862 | 2,718,634 | 8,364,726 | 13.34 |
| sF2 | 111,348,638 | 94,437,658 | 78,762,590 | 4,987,700 | 16,910,980 | 15.19 |
| sF3 | 80,824,593 | 61,321,111 | 52,127,848 | 2,868,193 | 19,503,482 | 24.13 |
| bO1 | 60,987,961 | 54,988,691 | 29,669,282 | 2,960,611 | 5,999,270 | 9.84 |
| bO2 | 83,483,600 | 74,676,180 | 63,194,578 | 4,419,900 | 8,807,420 | 10.55 |
| bO3 | 73,599,576 | 66,211,423 | 55,763,652 | 3,971,051 | 7,388,153 | 10.04 |
| sO1 | 62,449,538 | 53,494,629 | 43,577,282 | 3,214,019 | 8,954,909 | 14.34 |
| sO2 | 68,107,686 | 58,533,720 | 47,044,524 | 3,714,416 | 9,573,966 | 14.06 |

**Supplementary Table S2.**

Pearson’s correlations between replicates

| **Samples** | **R1 - R2** | **R2 - R3** | **R1 - R3** |
| --- | --- | --- | --- |
| bM | 0.8729 | 0.9772 | 0.8195 |
| sM | 0.9984 | 0.9877 | 0.9827 |
| bT | 0.9812 | 0.9934 | 0.9768 |
| sT | 0.9835 | 0.9251 | 0.9363 |
| bF | 0.9369 | 0.9991 | 0.9300 |
| sF | 0.9837 | 0.9781 | 0.9807 |
| bO | 0.9941 | 0.9975 | 0.9970 |
| sO | 0.9962 | – | – |

**Supplementary Table S3.**

Numbers of detected genes upon different RPKM threshold

| **Sample/Threshold** | **0** | **0.1** | **0.5** | **1** | **2** | **5** | **10** |
| --- | --- | --- | --- | --- | --- | --- | --- |
| Across 8 samples | 10,127 | 10,015 | 9,673 | 9,488 | **9,224** | 8,724 | 8,149 |
| bM | 9,672 | 9,507 | 9,047 | 8,725 | **8,280** | 7,405 | 6,446 |
| sM | 9,681 | 9,515 | 9,027 | 8,708 | **8,273** | 7,372 | 6,359 |
| bT | 8,806 | 8,649 | 8,314 | 8,064 | **7,757** | 7,254 | 6,603 |
| sT | 9,325 | 9,039 | 8,485 | 8,174 | **7,838** | 7,330 | 6,680 |
| bF | 9,275 | 9,123 | 8,529 | 8,103 | **7,573** | 6,753 | 5,855 |
| sF | 9,707 | 9,501 | 9,053 | 8,779 | **8,425** | 7,603 | 6,564 |
| bO | 8,913 | 8,419 | 7,782 | 7,433 | **7,016** | 6,350 | 5,745 |
| sO | 9,376 | 9,143 | 8,574 | 8,195 | **7,800** | 7,182 | 6,533 |

The threshold was set to RPKM > 2 in this study.

**Supplementary Table S4.**

**Top 100 highly transcribed genes**

| **Gene_ID** | **Average_RPKM** | **Product** |
| --- | --- | --- |
| Smp_131110.1 | 17275.63 | p14 |
| Smp_009580.1 | 12387.30 | ubiquitin |
| Smp_054160.1 | 7615.95 | Glutathione S-transferase 28 kDa (GST 28) (GST class-mu) |
| Smp_900020.1 | 6325.22 | NADH dehydrogenase subunit 6 |
| Smp_099870.1 | 5146.33 | eukaryotic translation elongation factor 1 alpha |
| Smp_103610.1 | 4582.56 | cathepsin B peptidase (C01 family) |
| Smp_105450.1 | 4529.73 | saposin containing protein |
| Smp_900010.1 | 4220.24 | cytochrome c oxidase subunit II |
| Smp_042160.2 | 4038.49 | aldolase |
| Smp_056970.1 | 4034.84 | glyceraldehyde 3 phosphate dehydrogenase |
| Smp_067060.1 | 3855.32 | cathepsin B peptidase (C01 family) |
| Smp_040130.1 | 3794.04 | cyclophilin |
| Smp_106930.1 | 3738.35 | heat shock protein 70 |
| Smp_900000.1 | 3715.34 | cytochrome c oxidase subunit I |
| Smp_082240.1 | 3714.94 | histone H3 |
| Smp_900050.1 | 3712.15 | NADH dehydrogenase subunit 5 |
| Smp_105420.1 | 3679.49 | Saposin,IPR008139 Saposin |
| Smp_900090.1 | 3663.37 | NADH dehydrogenase subunit 4 |
| Smp_025860.1 | 3424.47 | hypothetical protein |
| Smp_179300.3 | 3190.36 | cellular nucleic acid binding protein |
| Smp_155060.2 | 3173.29 | phosphatase 2a inhibitor i2pp2a |
| Smp_191910.1 | 3157.09 | Stress protein DDR48 |
| Smp_090120.1 | 3104.07 | alpha tubulin |
| Smp_072330.2 | 3090.12 | heat shock protein |
| Smp_067800.1 | 2951.25 | fibrillin 2 |
| Smp_194910.1 | 2884.88 | Saposin B domain containing protein |
| Smp_009310.1 | 2853.07 | plasminogen activator inhibitor 1 RNA binding |
| Smp_095360.1 | 2835.05 | fatty acid binding protein |
| Smp_132670.1 | 2797.75 | myosin regulatory light chain 2 smooth muscle |
| Smp_003770.1 | 2753.80 | Histone H1 delta |
| Smp_034840.2 | 2704.76 | 14-3-3 protein epsilon |
| Smp_009760.1 | 2687.06 | hypothetical protein |
| Smp_013200.2 | 2664.79 | large subunit ribosomal protein l10e |
| Smp_053390.1 | 2655.44 | histone H4 |
| Smp_046690.1 | 2643.60 | polyubiquitin C |
| Smp_066990.1 | 2432.91 | 40s rRNA protein, putative |
| Smp_211250.1 | 2430.06 | Ribosomal protein L34a |
| Smp_035200.4 | 2345.64 | hsp40 subfamily A members 124 |
| Smp_063300.1 | 2302.98 | 60S ribosomal protein L36 |
| Smp_084460.1 | 2247.31 | ribosomal protein L28 |
| Smp_026560.2 | 2201.09 | calmodulin |
| Smp_008070.1 | 2200.04 | thioredoxin |
| Smp_180000.1 | 2178.42 | ribosomal protein S9 |
| Smp_098960.1 | 2157.14 | 60S ribosomal protein L26 |
| Smp_900060.1 | 2126.86 | Cytochrome c oxidase subunit III |
| Smp_050280.1 | 2124.72 | ribosomal protein L31 |
| Smp_072900.1 | 2102.32 | hsp90 co chaperone (tebp) |
| Smp_213130.1 | 2087.99 | ribosomal protein S25 |
| Smp_035800.1 | 2074.48 | 60S ribosomal protein L37 |
| Smp_066940.1 | 2058.21 | 60S ribosomal protein L29 |
| Smp_030730.1 | 2045.48 | tubulin beta 2B chain |
| Smp_051260.1 | 2045.00 | 60S ribosomal protein L8 |
| Smp_075800.1 | 2004.53 | hemoglobinase (C13 family) |
| Smp_038560.2 | 1994.10 | Prothymosin alpha B |
| Smp_100090.1 | 1994.00 | high mobility group protein B1 |
| Smp_035790.1 | 1984.71 | 60S ribosomal protein L44 |
| Smp_010940.1 | 1886.98 | Acidic leucine rich nuclear phosphoprotein pp32a |
| Smp_017430.1 | 1877.76 | multivalent antigen sj gapdh; multivalent antigen sj fabp |
| Smp_017280.1 | 1876.46 | small subunit ribosomal protein S27Ae |
| Smp_201060.1 | 1868.44 | dynein light chain |
| Smp_007900.1 | 1866.13 | large subunit ribosomal protein 23 |
| Smp_044580.1 | 1820.91 | ribosomal protein L30 |
| Smp_029820.1 | 1777.93 | ribosomal protein, large P2 |
| Smp_001830.1 | 1759.81 | ribosomal protein L24 |
| Smp_031310.1 | 1732.69 | Ribosomal protein, Small subunit |
| Smp_038510.1 | 1708.99 | 60s ribosomal protein l6 |
| Smp_900070.1 | 1696.64 | cytochrome B |
| Smp_074470.1 | 1693.84 | Ribosomal protein, Small subunit |
| Smp_053830.1 | 1681.76 | 40S ribosomal protein S14 |
| Smp_146190.1 | 1674.99 | 40S ribosomal protein S21 |
| Smp_003410.1 | 1654.26 | 60S ribosomal protein L23a |
| Smp_200920.1 | 1650.62 | ribosomal protein l5 |
| Smp_063350.1 | 1650.39 | ribosomal protein L27 |
| Smp_900040.1 | 1646.74 | NADH dehydrogenase subunit 2 |
| Smp_045510.1 | 1642.12 | small ubiquitin modifier |
| Smp_013470.2 | 1637.71 | small subunit ribosomal protein S2e |
| Smp_074000.1 | 1630.83 | hypothetical protein |
| Smp_073880.1 | 1602.69 | 40s ribosomal protein s3a |
| Smp_022640.1 | 1601.83 | ribosomal protein L13 |
| Smp_054780.1 | 1594.40 | 40S ribosomal protein S8 |
| Smp_007300.1 | 1593.82 | coiled coil helix coiled coil helix |
| Smp_130100.1 | 1587.45 | saposin containing protein |
| Smp_161920.1 | 1587.06 | actin |
| Smp_055050.1 | 1585.46 | 40S ribosomal protein S17 |
| Smp_093440.1 | 1568.20 | ribosomal protein s5a |
| Smp_042090.1 | 1564.96 | 60S ribosomal protein L10a |
| Smp_050940.1 | 1538.29 | 60S ribosomal protein L11 |
| Smp_097800.3 | 1531.34 | cold shock domain protein A |
| Smp_900110.1 | 1514.87 | NADH dehydrogenase subunit 1 |
| Smp_080920.4 | 1504.17 | hypothetical protein |
| Smp_030370.1 | 1491.66 | calreticulin |
| Smp_130850.1 | 1484.08 | 40s ribosomal protein s15 |
| Smp_050390.1 | 1478.86 | aldehyde dehydrogenase |
| Smp_123270.1 | 1478.27 | Sj Ts1 |
| Smp_174950.1 | 1465.38 | ribosomal protein S19e |
| Smp_008545.1 | 1464.42 | heat shock protein 60 HSP60 |
| Smp_089570.1 | 1457.99 | stress associated endoplasmic reticulum protein |
| Smp_039400.1 | 1453.95 | eukaryotic translation initiation factor 5A |
| Smp_111340.6 | 1450.81 | hypothetical protein |
| Smp_139160.1 | 1429.03 | SmCL2 peptidase (C01 family) |

**Supplementary Table S5 (separate file)**

Differential gene expression analyses based on pairing, gender and tissue-origin.

Supplementary Table S6 (separate file)

Lists of differentially expressed genes (DEGs) by pairing.

Supplementary Table S7 (separate file)

KEGG pathway mapping for genes more abundantly transcribed in bO and sO, respectively.

Supplementary Table S8 (separate file)

List of genes from each category, including log_2_FC, FDR, and annotations.

Supplementary Table S9 (separate file)

Transcript occurrence with > 1.5-fold difference between bF and sF, but no significant difference between sF and sM.

Supplementary Table S10 (separate file)

List of 1,012 genes, whose transcript levels were found to be at least 1.5-fold higher in all gonads than in the worm counterparts, and among which, 235 are annotated as ‘hypothetical’ and do not show conserved domains.

**Supplementary Table S11.**

Comparison of the average transcript levels of stem cells- and neoblasts-associated genes

| **Sample** | **Average_all genes** | **Average_NSe genes** | **Fold difference** |
| --- | --- | --- | --- |
| bM | 110.2 | 131.1 | 1.19 |
| sM | 119.9 | 148.0 | 1.23 |
| bT | 89.4 | 154.0 | 1.72 |
| sT | 87.0 | 156.2 | 1.80 |
| bF | 144.9 | 196.5 | 1.36 |
| sF | 120.4 | 161.0 | 1.34 |
| bO | 120.9 | 288.3 | 2.38 |
| sO | 79.8 | 161.8 | 2.03 |

NSe, neoblast/sporocyst-enriched; bM, bisex males; sM, single-sex males; bT, testes from bisex males; sT, testes from single-sex males; bF, bisex females; sF, single-sex females; bO, ovaries from bisex females; sO, ovaries from single-sex females.

**Supplementary Table S12.**

Expression levels of neuropeptide precursor genes

| **Gene** | **bM** | **sM** | **bT** | **sT** | **bF** | **sF** | **bO** | **sO** |
| --- | --- | --- | --- | --- | --- | --- | --- | --- |
| Smp_042120.1 | 54.03 | 57.65 | 0.37 | 0.48 | 3.95 | 41.80 | 0.41 | 0.15 |
| Smp_044680.1 | 215.51 | 201.58 | 882.91 | 759.14 | 134.50 | 193.85 | 140.78 | 234.21 |
| Smp_052880.1 | 254.91 | 69.51 | 0.15 | 3.29 | 68.70 | 299.93 | 0.79 | 50.27 |
| Smp_056360.8 | 762.43 | 541.27 | 1,228.25 | 1,123.80 | 918.82 | 623.98 | 1,089.14 | 2,240.10 |
| Smp_070100.1 | 386.04 | 372.47 | 3.89 | 7.85 | 89.69 | 476.90 | 0.58 | 2.86 |
| Smp_088360.1 | 2.83 | 1.51 | 0.39 | 0.03 | 0.52 | 3.76 | 0.01 | 0.00 |
| Smp_136760.1 | 108.17 | 97.84 | 2.28 | 2.90 | 36.56 | 175.73 | 0.23 | 4.72 |
| Smp_138560.1 | 0.28 | 0.14 | 31.36 | 19.53 | 0.00 | 0.03 | 0.00 | 0.16 |
| Smp_142160.1 | 173.62 | 164.80 | 1.79 | 2.31 | 20.54 | 125.71 | 2.09 | 7.42 |
| Smp_150650.1 | 292.05 | 216.95 | 3.52 | 6.23 | 57.67 | 261.10 | 1.66 | 59.58 |
| Smp_153070.1 | 27.89 | 29.69 | 2.90 | 3.04 | 58.39 | 122.65 | 0.08 | 1.10 |
| Smp_154970.1 | 197.84 | 166.07 | 0.30 | 0.58 | 36.51 | 226.77 | 0.36 | 4.55 |
| Smp_159950.1 | 0.76 | 1.56 | 0.46 | 0.14 | 0.92 | 4.32 | 0.05 | 1.20 |

Numbers represent means of unnormalized RPKM values.

**Supplementary Table S13.**

List of 15 genes with potential house-keeping function

| **GeneID** | **Product** |
| --- | --- |
| Smp_005050.1 | Oxidoreductase HTATIP2 |
| Smp_008900.1 | eukaryotic translation |
| Smp_018760.1 | neutral alpha glucosidase AB |
| Smp_048940.1 | Vacuolar protein sorting associated protein |
| Smp_064020.4 | PAB dependent poly(A) specific ribonuclease |
| Smp_065110.1 | LETM1 and EF hand domain containing protein 1 |
| Smp_073940.1 | actin interacting protein 1 |
| Smp_104960.1 | syntaxin 6 |
| Smp_106260.1 | transcription factor iib |
| Smp_129570.1 | hypothetical protein |
| Smp_138760.1 | ADP ribose pyrophosphatase, mitochondrial |
| Smp_152080.1 | hypothetical protein |
| Smp_152510.1 | CWF19 protein 1 |
| Smp_153490.1 | GDP mannose 4,6 dehydratase |
| Smp_166290.1 | serine:threonine protein phosphatase 2A |

**Supplementary Table S14 (separate file)**

Comparison with previous microdissection study. This list contains transcripts that occurred at least 2-fold more abundant in bO versus bF, or in bT versus bM, as discovered by a previous microarray study based on laser microdissection ^17^ and the current whole-gonad RNA-seq approach.

**Supplementary References**

1. Chen, L. L., Rekosh, D. M. & LoVerde, P. T. *Schistosoma mansoni* p48 eggshell protein gene: characterization, developmentally regulated expression and comparison to the p14 eggshell protein gene. *Mol. Biochem. Parasitol.* **52,** 39–52 (1992).

2. deWalick, S. *et al.* The proteome of the insoluble *Schistosoma mansoni* eggshell skeleton. *Int. J. Parasitol.* **41,** 523–32 (2011).

3. Irla, M. *et al.* Spatial, a new nuclear factor tightly regulated during mouse spermatogenesis. *Gene Expr. Patterns* **3,** 135–138 (2003).

4. O’Connell, M. L., Cavallo, W. C. & Firnberg, M. The expression of CPEB proteins is sequentially regulated during zebrafish oogenesis and embryogenesis. *Mol. Reprod. Dev.* **81,** 376–87 (2014).

5. Wegner, M. SOX after SOX: SOXession regulates neurogenesis. *Genes Dev.* **25,** 2423–2428 (2011).

6. DeMarco, R. *et al.* Protein variation in blood-dwelling schistosome worms generated by differential splicing of micro-exon gene transcripts. *Genome Res.* **20,** 1112–21 (2010).

7. Esterlechner, J. *et al.* LIN9, a subunit of the DREAM complex, regulates mitotic gene expression and proliferation of embryonic stem cells. *PLoS One* **8,** e62882 (2013).

8. Skinner, D. E. *et al.* Vasa-Like DEAD-Box RNA Helicases of Schistosoma mansoni. *PLoS Negl. Trop. Dis.* **6,** e1686 (2012).

9. Beckmann, S. *et al.* *Schistosoma mansoni*: signal transduction processes during the development of the reproductive organs. *Parasitology* **137,** 497–520 (2010).

10. Buro, C. *et al.* Transcriptome analyses of inhibitor-treated schistosome females provide evidence for cooperating Src-kinase and TGFβ receptor pathways controlling mitosis and eggshell formation. *PLoS Pathog.* **9,** e1003448 (2013).

11. Dissous, C., Morel, M. & Vanderstraete, M. Venus kinase receptors: prospects in signaling and biological functions of these Invertebrate kinases. *Front. Endocrinol. (Lausanne).* **5,** 72 (2014).

12. Ganguly, R., Hong, C. S., Smith, L. G. F., Kornblum, H. I. & Nakano, I. Maternal embryonic leucine zipper kinase: key kinase for stem cell phenotype in glioma and other cancers. *Mol. Cancer Ther.* **13,** 1393–8 (2014).

13. Beckmann, S. *et al.* Characterization of the Src/Abl hybrid kinase SmTK6 of *Schistosoma mansoni*. *J. Biol. Chem.* **286,** 42325–42336 (2011).

14. Hahnel, S. *et al.* Gonad RNA-specific qRT-PCR analyses identify genes with potential functions in schistosome reproduction such as SmFz1 and SmFGFRs. *Front. Genet.* **5,** 1–15 (2014).

15. Hahnel, S., Lu, Z., Wilson, R. A., Grevelding, C. G. & Quack, T. Whole-organ isolation approach as a basis for tissue-specific analyses in *Schistosoma mansoni*. *PLoS Negl. Trop. Dis.* **7,** e2336 (2013).

16. Neves, R. H. *et al.* A new description of the reproductive system of *Schistosoma mansoni* (Trematoda: Schistosomatidae) analyzed by confocal laser scanning microscopy. *Parasitol. Res.* **95,** 43–9 (2005).

17. Nawaratna, S. S. K., McManus, D. P., Moertel, L., Gobert, G. N. & Jones, M. K. Gene Atlasing of digestive and reproductive tissues in *Schistosoma mansoni*. *PLoS Negl. Trop. Dis.* **5,** e1043 (2011).
